# Efficacy of probiotics on cognition, and biomarkers of inflammation and oxidative stress in adults with Alzheimer’s disease or mild cognitive impairment — a meta-analysis of randomized controlled trials

**DOI:** 10.18632/aging.102810

**Published:** 2020-02-15

**Authors:** Haoyue Den, Xunhu Dong, Mingliang Chen, Zhongmin Zou

**Affiliations:** 1 State Key Laboratory of Trauma, Burns and Combined Injury, Second Department of Research Institute of Surgery, Daping Hospital, Third Military Medical University (Army Medical University), Chongqing 400042, China; 2 Department of Chemical Defense, School of Military Preventive Medicine, Third Military Medical University (Army Medical University), Chongqing 400038, China; 3 Institute of Pathology and Southwest Cancer Centre, Southwest Hospital, Third Military Medical University (Army Medical University), Chongqing 400038, China

**Keywords:** probiotics, cognition, Alzheimer’s disease, mild cognitive impairment, meta-analysis

## Abstract

Probiotics are live microbes that confer health benefits to the host. Preliminary animal evidence supports the potential role of probiotics in ameliorating cognitive health, however, findings from clinical trials in Alzheimer’s disease (AD) or mild cognitive impairment (MCI) subjects are controversial. Thus, a meta-analysis is needed to clarify the efficacy of probiotics on cognition in AD or MCI patients. EMBASE, PubMed, Web of Science and Cochrane library were systematically searched and manually screened for relevant published randomized controlled trials (RCTs). Among the 890 citations identified, 5 studies involving 297 subjects met eligibility. There was a significant improvement in cognition (SMD = 0.37; 95% CI, 0.14, 0.61; *P* = 0.002; *I^2^* = 24%), while a significant reduction in malondialdehyde (SMD = −0.60; 95% CI, −0.91, −0.28; *P* = 0.000; *I^2^* = 0.0%) and high-sensitivity C-reactive protein (SMD = −0.57; 95% CI, −0.95, −0.20; *P* = 0.003; *I^2^* = 0.0%) post-intervention levels between the probiotics and control group. This meta-analysis indicated that probiotics improved cognitive performance in AD or MCI patients, possibly through decreasing levels of inflammatory and oxidative biomarkers. However, current evidence is insufficient, and more reliable evidence from large-scale, long-period, RCT is needed.

## INTRODUCTION

The incidence of Alzheimer’s disease (AD) is increasing globally and has reached the point of being a costly public health issue [1]. AD, featured by impaired cognition and memory loss, is hidden onset and possesses a long incubation period [2], during which mild cognitive impairment (MCI) constitutes the typical prodromal stage [3]. The *World Alzheimer Report 2019* [4] showed that 50 million people are living with dementia worldwide. Remarkably, there will be one new case every 3 seconds, and the number is predicted to be 152 million persons by 2050 [1, 4], resulting in a huge lifestyle and economic burden to both the patients and their families. The total estimated worldwide cost of dementia was US$1 trillion and will rise to US$2 trillion by 2030 [1, 4]. Unfortunately, there is currently no curative treatment for cognitive impairment and dementia [5].

The gut microbiota (GM) consists of a vast bacterial community that resides primarily in the lower gut and lives in a symbiotic relationship with the host [6]. Emerging experimental and clinical evidence has demonstrated the close interconnection between the gastrointestinal tract and the brain, known as the gut-brain axis [7]. Recently, the GM has been found to regulate brain development and behavior via the gut-brain axis, and this has been called the microbiota-gut-brain (MGB) axis [8]. It has been found that dysfunction in behavior and cognition is associated with GM dysbiosis [9–13]. Activation of gut inflammation has been regarded as a possible pathogenic cofactor in cognitive deterioration and dementia [14, 15]. Moreover, the most distinctive alterations in the GM of AD patients are decreased abundance of anti-inflammatory bacterial species (e.g. *Bifidobacterium breve*strain A1) and increased abundance of pro-inflammatory flora phyla (e.g. *Firmicutes* and *Bacteroidetes*) [16–18]. And restoring GM homeostasis could slow down the progression of AD [18, 19]. Therefore, the GM has been proposed as a key player in the pathogenesis of AD and might be a new potential therapeutic target for the prevention and treatment of AD [20, 21].

Probiotics are live microbes that confer health benefits to the host when administered in adequate amounts, possibly through their anti-inflammatory or anti-oxidative effects [22–24]. Recently, some probiotics have been shown to influence the central nervous system (CNS) and behavior via the MGB axis [25]. Moreover, eleven preclinical studies have shown that neither single strains nor multi-strain probiotics were beneficial for improving cognitive function in animal models [26]. These preclinical results have indicated that probiotics might be an effective dietary intervention to ameliorate age-associated cognitive deficits. Nevertheless, findings from available clinical trials focusing on the effects of probiotics in patients with AD or MCI are inconsistent [27–31]. Additionally, the previous relevant reviews are focused on the effect of probiotics on neurodegenerative and neurodevelopmental disorders in both animal models and human trials. And they found that probiotics showed efficacy in improving psychiatric disorder-related behaviors including anxiety, depression, autism spectrum disorder, and memory abilities [26, 32]. However, the evidence for the effects of psychobiotics on mental and neurological conditions/disorders remains limited. Further studies of psychobiotics are needed in order to determine their effectiveness and mechanism as treatments for various psychiatric disorders. Accordingly, this meta-analysis was the first to quantitatively examine the potential effect of probiotics on cognitive performance and inflammatory and oxidative biomarkers, which might be related to the underlying mechanisms in AD or MCI subjects.

## RESULTS

### Literature search and screening

**Figure 1:**
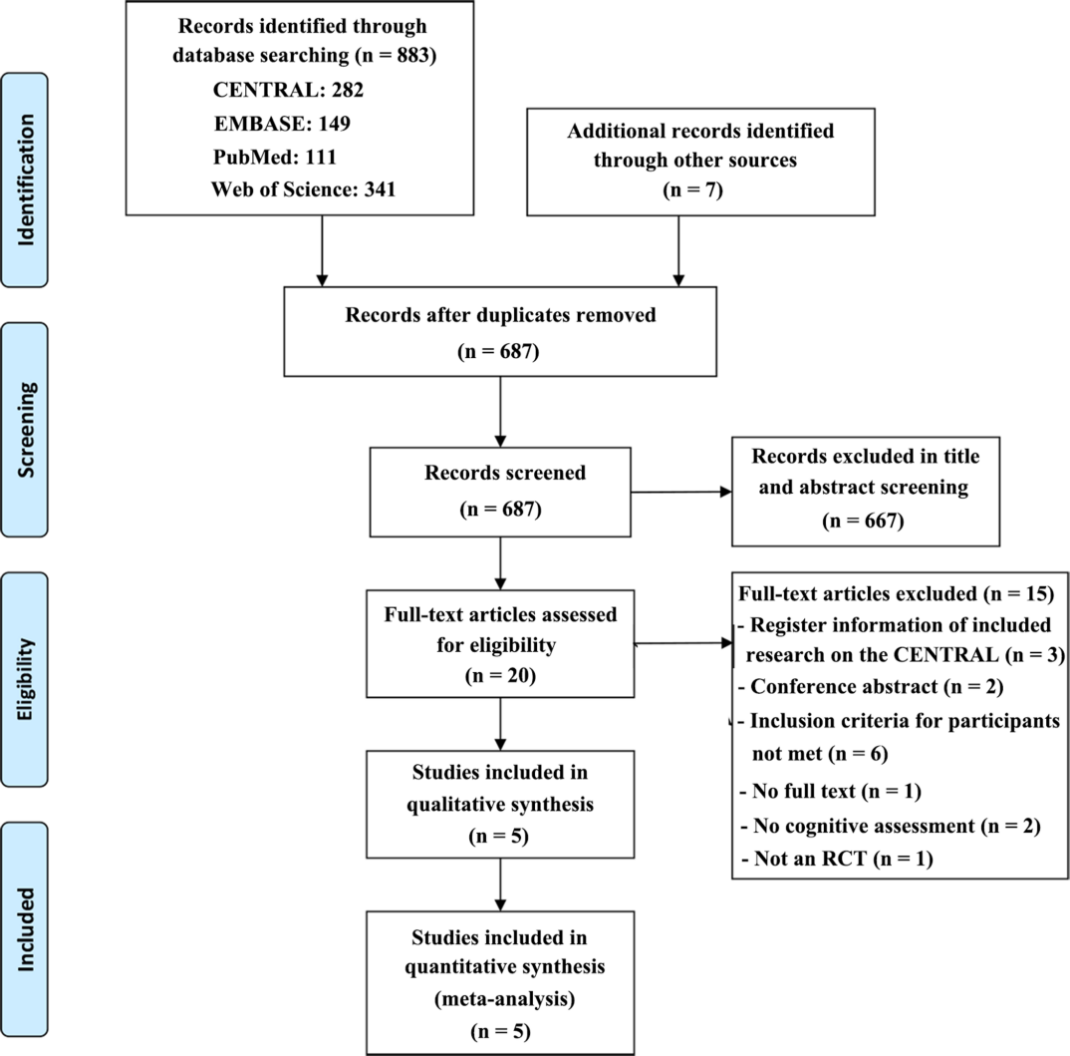
**PRISMA flow diagram of the literature search and abstraction process.**

A total of 883 records were obtained after the initial search of the electronic databases, and 7 studies were identified through manual searching of the reference lists of relevant published reviews (for a detailed description of the literature search and results, please see the supplementary materials). Of these, 203 trials were removed as duplicates, and 667 publications were excluded after screening the title and abstract. The remaining 20 articles were scrutinized with a full-text screening, of which 15 were excluded for reasons detailed in the PRISMA flow chart (Figure 1). Then, 5 studies were considered eligible and finally included in the quantitative meta-analysis.

### Study characteristics

As shown in Table 1, the publication years of the five included studies ranged from 2016 to 2019, with an aggregated sample of 297 individuals. All studies were randomized, double-blind and controlled trials. Among which, three studies [27–29] recruited subjects diagnosed with AD and another two [30, 31] included MCI adults. Most studies included a higher ratio of women except for two studies (one [30] had a balanced proportion and the other [28] did not report the sex ratio of the recruited subjects).

**Table 1:** Main characteristics of the included studies.

**Study**	**Study design**	**Participants**						**Intervention**			**Outcome assessments**		**Main findings**
		**Sample size**	**Type (diagnostic criteria)**	**Age (M±SD)**		**Sex ratio (M/F)**		**Type of strains**	**Duration (weeks)**	**Dosage**	**Cognitive outcomes**	**Inflammatory/ oxidative biomarkers**	
				**PRO**	**CON**	**PRO**	**CON**						
Elmira Akbari (2016)	Randomized, double-blind, controlled trial	60	AD (NINCDS- ADRDA criteria)	77.67 ± 2.62	82.00 ± 1.69	6/24	6/24	Multiple (*Lactobacillus acidophilus*, *Lactobacillus* *casei*, *Bifidobacterium bifidum, Lactobacillus fermentum*)	12	8×10^9^ (CFU/ g)	MMSE	TAC GSH MDA hs-CRP NO	Probiotic consumption for 12 weeks positively affected cognitive function and some metabolic statuses in the AD patients
Omid Reza Tamtaji (2018)	Randomized, double-blind, controlled trial	90	AD (NINCDS- ADRDA criteria)	76.2 ± 8.1	78.8 ± 10.2			Multiple (*Lactobacillus acidophilus, Bifidobacterium bifidum, Bifidobacterium longum*)	12	6×10^9^ (CFU/ day)	MMSE	TAC GSH MDA hs-CRP NO	Probiotic and selenium co-supplementation for 12 weeks to patients with AD improved cognitive function and some metabolic profiles.
Azadeh Agahi (2018)	Randomized, double-blind, controlled trial	48	AD (NINCDS- ADRDA criteria)	79.70 ± 1.72	80.57 ± 1.79	7/18	10/13	Multiple (*Lactobacillus fermentum, Lactobacillus* *plantarum, Bifidobacterium lactis Lactobacillus acidophilus,* *Bifidobacterium bifidum, Bifidobacterium longum*)	12	3×10^9^ (CFU/ day)	TYM	TAC GSH MDA NO	Cognitive and biochemical indications in the patients with severe AD were insensitive to the probiotic supplementation.
Y. Kobayashi (2019)	Randomized, double-blind, controlled trial	121	Subjective memory complaints (MMSE, 22-27)	61.5 ± 6.83	61.6 ± 6.37	30/31	30/30	Sole (*Bifidobacterium breve* A1)	12	>2.0×10^10^ (CFU/ day)	MMSE	hs-CRP	No significant intergroup difference was observed in terms of changes in scores from the baseline scores
		44	MCI (RBANS <41)					Sole (*Bifidobacterium breve* A1)	12	>2.0×10^10^ (CFU/ day)	MMSE		Significant difference between *B. breve* A1 and placebo groups in terms of MMSE total score in the subjects with MCI
Yun-Ha Hwang (2019)	Multi-center, randomized, double-blind, controlled trial	100	MCI (DSM-5)	68.0 ± 5.12	69.2 ± 7.00	20/30	14/36	Sole (*Lactobacillus plantarum* C29)	12	>1.0 × 10^10^ (CFU/ day)	VLT ACPT DST		DW2009 can be safely administered to enhance cognitive function in individuals with MCI.

Abbreviations: PRO, probiotics group; CON, control group; CFU, colony-forming units; AD, Alzheimer’s disease; MCI, mild cognitive impairment; NINCDS-ADRDA criteria, National Institute of Neurological and Communicative Disorders and Stroke (NINCDS) and the Alzheimer’s Disease and Related Disorders Association (ADRDA); MMSE, Mini-Mental State Examination; RBANS, Repeatable Battery for the Assessment of Neuropsychological Status; TAC, total anti-oxidant capacity; GSH, total glutathione; MDA, malondialdehyde; hs-CRP, high-sensitivity C-reactive protein; NO, nitric oxide; DSM-5, Diagnostic and Statistical Manual of Mental Disorders, 5^th^ edition; VLT, verbal learning test; ACPT, auditory continuous performance test; DST, digit span test; DW2009, *Lactobacillus plantarum* C29-fermented soybean.

The intervention duration of the involved studies was all 12 weeks. Two studies [30, 31] used a sole strain of probiotics, while the other three studies applied multiple strains. The study from Azadeh Agahi et al. [29] had two active probiotics groups which were regarded as one group for the present analysis. All studies used a matched placebo that was indistinguishable from the probiotics in terms of packaging, form, appearance, size, taste, smell, and so forth.

With regard to the main findings, three studies [27, 28, 31] found significant difference in improving cognition between the probiotics and control groups, whereas one study [29] found no significant difference, and another study [30] reported mixed findings. Similarly, the results regarding changes in various inflammatory and oxidative metabolites were inconsistent across studies.

### Risk of bias assessment

**Figure 2:**
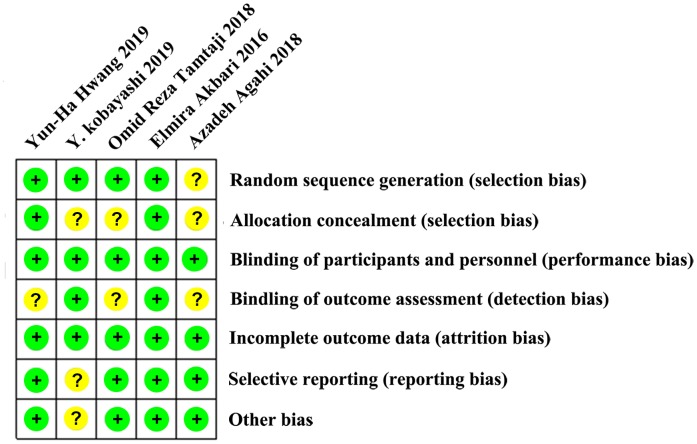
**Summary of risk of bias assessment: judgments of the review authors on each risk of bias item for the included studies (n = 5).** One study was rated as “low risk of bias”, and the other four were assessed as “moderate risk of bias.” No study was judged as “high risk of bias”.

Although all five included studies were RCT designs, one study [29] failed to provide information on the random sequence generation, and three studies [28–30] had no information on allocation concealment procedures. All studies described the blindness of the participants and implementing personnel, whereas only two studies [27, 30] reported the blindness of outcome assessments. No risk of incomplete outcome data was found for any of the recruited studies. Overall, the assessment of bias generally reported a low to moderate risk of bias across all domains. The risks of bias assessment across the recruited studies are summarized in Figure 2.

### Meta-analysis: Main results

**Figure 3:**
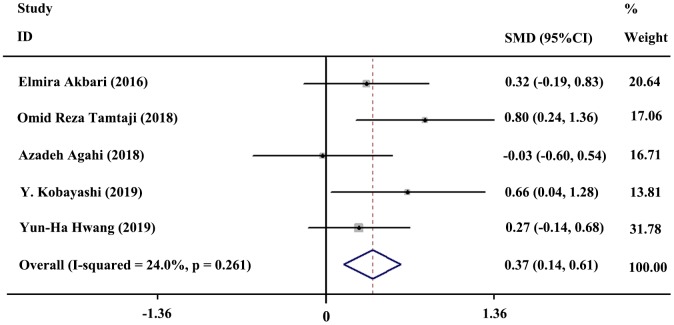
**Forest plot showing the standardized mean difference (SMD) in cognitive enhancement, comparing the probiotics group versus the control group.** Weights were assigned according to the number of subjects and SD using STATA 12. A fixed-effects model was applied to the meta-analysis. The sizes of the data markers represent the weight of each study, and the diamond indicates the overall estimated effect.

Five studies included 154 subjects in the probiotics group and 143 subjects in the control group. A fixed-effects model was selected for quantitative synthesis. In general, the result of meta-analysis revealed a significant difference between the probiotics and control group regarding improvement in cognition (SMD = 0.37; 95% CI, 0.14, 0.61; *P* = 0.002; *I^2^*= 24%). The forest plot of the meta-analysis is shown in Figure 3.

**Figure 4:**
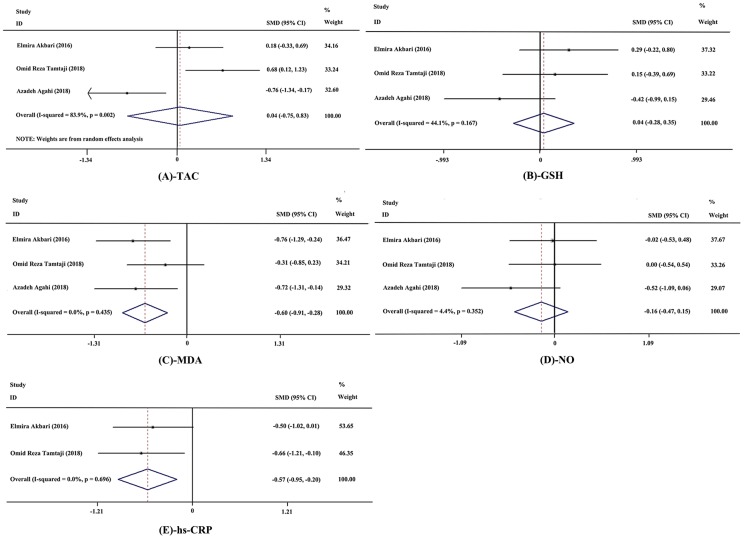
**Forest plot showing the effects in the probiotics group versus the control group on biomarkers of inflammation and oxidative stress.** (**A**) Meta-analysis of the effects of probiotics on total anti-oxidant capacity (TAC). (**B**) Meta-analysis of the effects of probiotics on total glutathione (GSH). (**C**) Meta-analysis of the effects of probiotics on malondialdehyde (MDA). (**D**) Meta-analysis of the effects of probiotics on nitric oxide (NO). (**E**) Meta-analysis of the effects of probiotics on high-sensitivity C-reactive protein (hs-CRP). Weights were assigned according to the number of subjects and SD using STATA 12. A random-effects model was applied to the meta-analysis of TAC, while other biomarkers used a fixed-effects model. The sizes of data markers represent the weight of each study, and the diamonds indicate the overall estimated effect. SMD, standardized mean difference.

Meanwhile, fixed-effects models were applied to meta-analyses of malondialdehyde (MDA), high-sensitivity C-reactive protein (hs-CRP), total glutathione (GSH) and nitric oxide (NO) on account of acceptable heterogeneity among studies, while a random-effects model was selected for analysis of total anti-oxidant capacity (TAC). The results showed significant decrease in levels of MDA (SMD = −0.60; 95% CI, −0.91, −0.28; *P* = 0.000; *I^2^* = 0.0%; n = 3 studies; n = 82 subjects) and hs-CRP (SMD = −0.57; 95% CI, −0.95, −0.20; *P* = 0.003; *I^2^* = 0.0%, n = 2 studies, n = 57 subjects). And no significant differences were observed in TAC (SMD = 0.04; 95% CI, −0.75, 0.83; *P* = 0.919; *I^2^* = 83.9%, n = 3 studies; n = 82 subjects), GSH (SMD = 0.04; 95% CI, −0.28, 0.35; *P* = 0.822; *I^2^* = 44.1%; n = 3 studies; n = 82 subjects), and NO (SMD = −0.16; 95% CI, −0.47, 0.15; *P* = 0.316; *I^2^* = 4.4%; n = 3 studies; n = 82 subjects) between the probiotics and control group. The forest plots of these meta-analyses are shown in Figure 4.

### Subgroup analyses

A total of three subgroup analyses were conducted, and the results were summarized in Table 2 (for forest plots of the subgroup analyses, please see the supplementary materials). The heterogeneity obviously declined only in the subgroup analysis of “MMSE versus non-MMSE” after stratification by cognitive rating scales. Additionally, the meta-analysis results from the subgroups for disease type (AD versus MCI) and strains of flora (multiple versus sole) were consistent with the overall pooled results, while there was no effect on cognition in the subset of studies with the non-MMSE rating scales (SMD = 0.16; 95% CI: −0.17, 0.50; *P* = 0.33; *I^2^* = 0.0%).

**Table 2:** Summary of meta-analysis and subgroup analyses on cognition.

**Outcome or Subgroup**	**No. of trials**	**Participants**	**Estimated Effect**	**Test of heterogeneity**		***P^2^***
				***P^1^***	***I^2^* (%)**	
1.0 Probiotics versus control group	5	297	0.37 (0.14, 0.61)	0.26	24	0.002
1.1 Subgroup by type of disease						
1.1.1 AD	3	161	0.36 (0.05, 0.68)	0.12	52.2	0.023
1.1.2 MCI	2	136	0.39 (0.04, 0.73)	0.30	6.4	0.028
1.2 Subgroup by cognitive rating scale						
1.2.1 MMSE	3	157	0.57 (0.25, 0.89)	0.44	0	0.001
1.2.2 Non-MMSE	2	140	0.16 (-0.17, 0.50)	0.41	0	0.33
1.3 Subgroup by strains of flora						
1.3.1 Multiple	3	161	0.36 (0.05, 0.68)	0.12	52.2	0.023
1.3.2 Sole	2	136	0.39 (0.04, 0.73)	0.30	6.4	0.028

Note: *P^1^* for heterogeneity: *P* < 0.1 was considered to indicate significant heterogeneity across studies. *I^2^* for heterogeneity: *I^2^* > 50% was considered to indicate significant heterogeneity across studies. *P^2^* for meta-analysis: *P* < 0.05 was considered to indicate a significant effect of probiotics on cognition by using a fixed-effects model.

### Publication bias assessment and sensitivity analysis

We quantitatively assessed the publication bias through Egger's test and Begg's test. The results demonstrated no significant publication bias on cognition (Egger's test: *P* = 0.54, Begg's test: *P* = 0.81; see supplementary materials). The sensitivity analysis was performed to test the reliability of the meta-analysis results by omitting studies one by one. Systematically removing each trial did not significantly influence the overall effects of probiotics on cognition (see supplementary materials). Thus, the findings between the probiotics and control groups regarding cognitive enhancement were considered reliable.

## DISCUSSION

To the best of our knowledge, this study is the first meta-analysis to elucidate the pronounced beneficial effects of probiotics on improving cognition in individuals with AD or MCI, which might be attributed to the effective decrease in some inflammatory and oxidative related biomarkers (MDA and hs-CRP).

Over the past decades, many studies have focused on effective pharmaceutical strategies to control AD; however, none have yet yielded a satisfying therapy. Recently, it has been demonstrated that modification of lifestyle factors such as dietary patterns plays an important role in alleviating cognitive decline [33]. This indicates that it is imperative to explore potential dietary interventions that may ameliorate age-associated cognitive deficits. Probiotics intervention could be a novel and feasible dietary strategy for managing dementia and other related diseases [34]. Remarkably, our findings provide evidence that relatively low-cost, widely available, and well-tolerated probiotics could be potential candidates for the control or prevention of AD and MCI. However, the specific underlying mechanisms remain unknown.

Although the exact pathogenesis of AD is not completely understood, accumulating evidence suggests that the oxidative stress and inflammatory pathways may play a critical role in the underlying mechanisms of cognitive deficits and AD [35]. Increased neuroinflammation and oxidative stress are frequently observed complications in participants with MCI and AD [36–38]. Amyloid-beta peptide (Aβ) oligomers has been considered as the major pathogenic factor associated to AD, which tend to accumulate extracellularly as amyloid deposits [39]. Neuroinflammation in AD is thought to be triggered by Aβ and/or by the substances released by dying neurons, causing microglia activation [40, 41]. Activated microglia triggers the recruitment and proliferation of astrocytes that actively bolster the inflammatory response to extracellular Aβ deposits. This neuroinflammatory component of AD is further characterized by a local cytokine (e.g. TNF-α, IL-1β, IL-6) accumulation mediating acute-phase response, activation of the complement cascade and induction of inflammatory enzyme systems such as inducible nitric oxide synthase (iNOS) and COX-2 [42, 43]. Meanwhile, Aβ is also shown to trigger oxidative stress by increasing ROS production through damage in mitochondrial structure and function [44, 45], or the activation of NADPH oxidase in microglia and astrocytes [46, 47], which in turn promotes further Aβ production, triggering a vicious cycle. All these factors, either alone or in concert, can contribute to neuronal dysfunction and death that occur in AD. Accordingly, huge numbers of potential anti-inflammatory and anti-oxidative agents have been explored to develop effective therapeutic strategies for AD.

GM has been reported to play an important role in the pathogenesis of AD through the MGB axis [21], indicating that restoring GM homeostasis may induce beneficial effects on the progression of AD [48]. Probiotics are live microorganisms intended to provide health benefits, generally by improving or restoring the GM [22–24, 49]. Moreover, Athari Nik Azm et al. [50] pointed out that reduction in the number of amyloid plaques, inflammation and oxidative stress were observed in an Alzheimer-probiotics group. Kobayashi et al. [51] also revealed that probiotics administration suppressed hippocampal gene expression of inflammation and oxidative stress related genes in an AD mouse model. These results suggest that probiotics might exert health benefits through their anti- inflammatory and anti-oxidative effects. Herein, we found that some inflammatory and oxidative biomarkers (hs-CRP and MDA) were significantly decreased by probiotics, which might be related to the underlying mechanisms of their beneficial effects in AD or MCI subjects. Meanwhile, some inflammatory and oxidative biomarkers (TAC, GSH, and NO) were not significantly different in the meta-analysis. Apart from diverse interventions conditions, such as strains of flora, total intervention duration, dosage, and so forth, it is worthwhile to infer that inflammatory and oxidative stress pathways are not specific to AD and MCI, but potentially play an important role in various kinds of diseases, especially age-related diseases [11]. Additionally, the inflammatory and oxidative biomarkers enrolled in the current study were only a part of the pathophysiology in AD and MCI patients. Therefore, more comprehensive inflammatory and oxidative metabolites need to be tested in future clinical trials in order to clarify the exact mechanism of the beneficial effect of probiotics on AD and MCI patients.

Furthermore, a systematic review reveals that neither single nor multi-strain probiotics are beneficial for improving cognitive function according to eleven animal studies [26]. Many studies demonstrate that probiotics, such as *Bifidobacterium* and *Lactobacillus* strains, ameliorate cognitive and memory deficits in animal models of AD [50, 52, 53]. In the current study, the strains used in the involved clinical trials were also from *Bifidobacterium* and *Lactobacillus* (Table 3). We found that sole or multi-strain probiotics were both beneficial for improving cognitive function, suggesting that *Bifidobacterium* and *Lactobacillus* strains would be the most potential candidates. However, AD patients were insensitive to a mixture of six type of strains in the study from Azadeh Agahi et al. [29], indicating that the effectiveness of probiotics may be influenced by multifactors such as the severity of patients, proportion of each strains, dosage and so forth.

**Table 3:** Components of the probiotics in each RCTs.

**Type of strains**	**Elmira Akbari (2016)**	**Omid Reza Tamtaji (2018)**	**Azadeh Agahi (2018)**	**Y. Kobayashi (2019)**	**Yun-Ha Hwang (2019)**
*Bifidobacterium bifidum*	√	√	√		
*Bifidobacterium breve* A1				√	
*Bifidobacterium lactis*			√		
*Bifidobacterium longum*		√	√		
*Lactobacillus acidophilus*	√	√	√		
*Lactobacillus* *casei*	√				
*Lactobacillus fermentum*	√		√		
*Lactobacillus plantarum*			√		√

Note: √ represents containing this type of strain in the component of probiotics.

Given the differences exist across species, the appropriate dosing of probiotics should be demonstrated for the specific target host [54]. The definition of probiotics requires the administration of an “adequate amount” in order to obtain a health benefit. However, the definition does not specify the size of this “adequate” dose [55, 56]. The commonly dose of probiotics used are ranging from 10^8^ to 10^11^ CFU in related studies. Some researches [57–59] appear to support a dose-response for probiotics in reducing the risk of antibiotic associated diarrhoea, suggesting that a dose greater than 10^10^ CFU is most effective. In contrast, there is no dose-response for probiotics in *Clostridium difficile* associated diarrhoea, necrotising enterocolitis, atopic dermatitis and slow intestinal transit [60–67]. Compiled evidence indicates that the effective dose of probiotics is influenced by a multitude of variables, including health endpoint, the specific probiotic used, delivery vehicle and route of administration [68]. Therefore, it seems difficult to epitomize one optimal dose for probiotic intervention. At present, we failed to provide a dose-response of probiotics in ameliorating cognitive deficits for the information on the intervention dose of probiotics was not enough in the involved studies. Nevertheless, the absence of evidence for a dose-effect does not imply evidence of absence of a dose-effect [67]. More reliable evidence from various dosages especially outside the common concentration (10^8^ ~ 10^11^ CFU) of probiotics trials is needed.

Additionally, the safety of probiotics should also be considered. People have been eating fermented foods for centuries without suffering any harmful side effects. Probiotics are generally accepted in the medical community as a harmless and very efficient, natural remedy. However, the United States Food and Drug Administration requires phase I safety studies for probiotics when the intended use of the product is as a drug. Researchers [69] have performed 10^8^ to 10^11^ CFU/day doses of *Bifidobacterium animalis*subsp *lactis* (BB-12) and *Lactobacillus paracasei* subsp *paracasei* (CRL-431) in healthy young adults, and found that the increasing doses of probiotic bacteria were well tolerated and no volunteers reported adverse side effects during the intervention. Moreover, Patricia L. Hibberd et al. [70] reported that *Lactobacillus rhamnosus* GG ATCC 53103 (LGG)(1×10^10^ CFU), one of the most studied probiotic, is safe and well tolerated in healthy adults aged 65 years and older. Among the five studies included in our present study, two [28, 29] did not report on the incidence of side effects; two [27, 30] reported no side effects and only one [31] reported mild adverse events (e.g. stomach aches, headaches, gastritis, erectile dysfunction). These results indicate that probiotics are considered generally safe to consume, but may cause bacteria-host interactions and unwanted side effects in rare cases. Accordingly, the *Bifidobacterium* and *Lactobacillus* strains used in the involved studies were safe and well tolerated, however, the specific strain still need phase I safety studies before using as a drug.

The main limitations are as follows: 1) First, the cognitive rating scales varied across the recruited studies. The widely accepted cognitive rating scale, the MMSE, was selected in three studies [27, 28, 30], and one trial chose the Test Your Memory (TYM) test. Remarkably, AD patients assessed by the MMSE [27, 28] revealed a positive effect of the probiotics, while the TYM test in patients with AD found the opposite result. The main reason for this discrepancy may arise from the difference in the outcome indexes among studies; furthermore, the TYM test has been reported to be more sensitive in detecting dementia than the MMSE [71]. In addition, Yun-Ha Hwang et al. [31] assessed three cognitive domains combined using the verbal learning test (VLT), auditory continuous performance test (ACPT), and digit span test (DST). Cognitive domains of this combined one are incomprehensive enough compared to the MMSE or TYM. The subgroup analysis showed that cognitive rating scales, i.e., “MMSE versus non-MMSE”, could partly explain the source of heterogeneity, and the imbalanced assessment methods may have discounted the meta-analysis outcome. 2) Second, the inclusion criteria for AD patients were all in accordance with the NINCDS-ADRDA criteria. However, the criteria for recruiting MCI subjects were different: Y. Kobayashi et al. [30] discriminated MCI and normal individuals using the Repeatable Battery for the Assessment of Neuropsychological Status (RBANS), while Yun-Ha Hwang et al. [31] diagnosed MCI according to the Diagnostic and Statistical Manual of Mental Disorders, 5^th^ edition (DSM-5). This may have generated low comparability of these two trials attributable to inconsistent inclusion criteria. 3) Third, three of the five clinical trials used in the cognitive meta-analysis are from the same region. Though the participants’ characteristics, intervention conditions, cognitive rating scales were varied from each other and the main findings were inconsistent. There is still concern that the relatively low heterogeneity might be partially attributed to the clinical trials from the same region. Additionally, two of the three studies also had concerns regarding allocation bias and evaluation bias. These limitations might discount the outcomes of our meta-analysis. 4) Fourth, some other key variables, including the age, sex, and BMI of the samples, specific strain of the flora, dosage of probiotics, and drug form, may have exerted an influence on the results, but the relevant information obtained on the results was limited in this study. Moreover, possible dose-response of probiotics in ameliorating cognitive deficits also should be further explored. 5) Last but not least, we concluded that the potential mechanism was partially related to the anti-inflammatory and anti-oxidative property of probiotics in only 12 weeks study duration. However, many clinical trials of AD drugs (i.e, β-amyloidantibody, β-secretase inhibitors, tau aggregation inhibitor), long-term interventions of several years have failed [72–74]. The beneficial efficacy of probiotics for AD and MCI in only 12 weeks study duration might be attributed to multi-directional mechanism regulation, including alterations in the levels of certain neurotransmitters, increasing neuroprotective molecules, such as brain-derived neurotrophic factor, reduction of inflammation and so forth [21, 75, 76]. In addition, there might be existence of sponsor bias in part of the included studies. Therefore, longer study durations are needed to determine the overall net benefits of probiotics. With a view to the above limitations, future RCTs that control these confounding factors are needed to clarify the moderators of probiotics effects on cognition.

## CONCLUSIONS

The meta-analysis indicated that probiotics consumption enhanced cognition in subjects with AD or MCI, possibly through decreasing the levels of inflammatory and oxidative biomarkers. However, the current RCT evidence is insufficient and limited, the results should be cautiously interpreted, and more reliable evidence from large-scale, long-period, randomized, controlled trials is needed.

## METHODS

### Literature search

A literature search was conducted according to the Preferred Reporting Items for Systematic Reviews and Meta-Analyses (PRISMA) guidelines. The preliminary search was performed through the EMBASE, PubMed, Web of Science and the Cochrane Central Register of Controlled Trials (CENTRAL) electronic databases without time restrictions, up to June 10, 2019. A snowball search was manually carried out by searching reference lists from relevant published reviews and the retrieved papers. We searched the first-group-term (AD or MCI-related term) with “OR”. Then used the same way for the second-group-term (human-related term) search and the third-group-term (probiotic-related term) search, followed by combing the number of records generated from the three groups of search term with “AND”. The following terms were searched in “all fields” for each electronic database: (Alzheimer’s disease OR dementia OR mild cognitive impairment OR cognitive dysfunction OR cognitive defect OR cognition OR memory OR mental capacity) AND (adult OR human) AND (probiotic OR yeast OR yoghurt OR fermented product OR lactobacillus OR bifidobacterium OR fermented dairy product OR synbiotics OR cultured milk products). Articles were limited to randomized controlled trials (RCTs).

### Study selection

Eligible studies had to meet the following criteria: (1) The study was an RCT and published in peer-reviewed journal in English; (2) Adult human participants who had a diagnosis of AD or MCI (aged over 18 y); (3) Significant difference in form, appearance, taste, and smell of probiotics and placebo should not occur at baseline; (4) Any validated measure of cognitive assessment was acceptable; (5) Continuous data of cognitive outcomes, inflammatory and oxidative biomarkers at baseline and post-intervention, or the change from baseline, and the number of participants at baseline and post-intervention were reported or could be calculated from the data reported in the article.

Studies were excluded if they met any one of the following criteria: (1) The publications were abstracts, reviews, conference papers, study protocols, cross-sectional studies, nonhuman (in vitro and animal) studies or papers that did not report on any outcome of interest; (2) No post-intervention or change from baseline on scale scores of cognition was reported, and these data could not be calculated based on the information in the article; (3) The study reported on a sample that overlapped the sample in another study. In this case, only the study with the larger sample size was included.

Articles were initially and independently screened for eligibility by two investigators based on titles and abstracts. Duplicate and irrelevant papers were excluded. For the relevant candidates, the full articles were retrieved, reviewed and the references of each document were checked to find out potential candidates. Disagreements were resolved by discussion between the two researchers, or with a third reviewer.

### Data extraction

Data were extracted independently by two authors (H-Y.D, M-L.C), using a predetermined form in accordance with the guidance of the Cochrane Handbook for Systematic Reviews of Interventions. Parameters collected including basic information of the RCTs (first author, published year, and study design), characteristics of the participants (sample size, disease type, age, and sex ratio), intervention-related variables (type of strains, duration, and dosage), outcome assessments (cognitive outcomes, inflammatory and oxidative biomarkers) and the main findings of each included study. To perform the meta-analysis, the following data of outcome assessments were extracted: the mean change score along with the associated variance (standard deviation [SD] or standard error of the mean [SEM]). When change scores were not available, the scores (mean±SD or mean±SEM) and the number of participants at baseline and post-intervention were extracted.

### Risk of bias assessment

All studies were independently assessed for risk of bias by two authors, with disagreements resolved by discussion to reach consensus. The tool of the Cochrane Handbook for Systematic Reviews of Interventions was used, which included criteria for the following six aspects: random sequence generation, allocation concealment, blindness of participants and personnel, blindness of outcome assessment, incomplete outcome data, selective reporting, and other sources of bias. The results of the risk of bias assessment were pooled into Revman 5.3, and a “summary of risk of bias assessment” table was generated.

### Statistical analysis

The primary outcomes of this study were the standardized mean differences (SMDs) of change from baseline between probiotics and placebo group. The SMD was tested by a Z statistic, and a two-tailed *P* < 0.05 was regarded as statistically significant. The inter-study heterogeneity was examined by chi-square (*χ2*) statistics and *I^2^* statistics. The heterogeneity among the different studies was considered high if *P* < 0.1 for the *χ2* statistic or *I^2^* > 50% [77]. SMDs were calculated by fixed-effects or random-effects models. A sensitivity analysis was conducted to test the reliability of the findings using the leave-one-out method, while publication bias was assessed by Egger's test and Begg's test. Subgroup analyses were performed to examine the possible source of heterogeneity within these studies, and the subtypes involved cognitive rating scales (Mini-Mental State Examination, MMSE, versus non-MMSE), type of disease (AD versus MCI) and strains of flora (multiple versus sole). The statistical analyses of forest plots, sensitivity analysis, Egger's test, and Begg's test were performed in STATA software (version 12; StataCorp), while Revman 5.3. was used to generate the summary of risk of bias assessment.

## Supplementary Material

Supplementary Materials

Supplementary Table 1

## References

[1] ADI. World Alzheimer Report 2018: The state of the art of dementia research: New frontiers. An Analysis of Prevalence, Incidence, Cost and Trends. London: Alzheimer's Disease International; 2018.

[2] Deng H, Mi MT. Resveratrol Attenuates Aβ25-35 Caused Neurotoxicity by Inducing Autophagy Through the TyrRS-PARP1-SIRT1 Signaling Pathway. Neurochem Res. 2016; 41:2367–79. 10.1007/s11064-016-1950-9 27180189

[3] Gauthier S, Reisberg B, Zaudig M, Petersen RC, Ritchie K, Broich K, Belleville S, Brodaty H, Bennett D, Chertkow H, Cummings JL, de Leon M, Feldman H, et al, and International Psychogeriatric Association Expert Conference on mild cognitive impairment. Mild cognitive impairment. Lancet. 2006; 367:1262–70. 10.1016/S0140-6736(06)68542-5 16631882

[4] ADI. World Alzheimer Report 2019: Attitudes to dementia. London: Alzheimer's Disease International; 2019.

[5] Manayi A, Saeidnia S, Gohari AR, Abdollahi M. Methods for the discovery of new anti-aging products—targeted approaches. Expert Opin Drug Discov. 2014; 9:383–405. 10.1517/17460441.2014.885014 24494592

[6] La Rosa F, Clerici M, Ratto D, Occhinegro A, Licito A, Romeo M, Iorio CD, Rossi P. The Gut-Brain Axis in Alzheimer's Disease and Omega-3. A Critical Overview of Clinical Trials. Nutrients. 2018; 10. 10.3390/nu10091267 30205543 PMC6164598

[7] Pistollato F, Sumalla Cano S, Elio I, Masias Vergara M, Giampieri F, Battino M. Role of gut microbiota and nutrients in amyloid formation and pathogenesis of Alzheimer disease. Nutr Rev. 2016; 74:624–34. 10.1093/nutrit/nuw023 27634977

[8] Mayer EA, Tillisch K, Gupta A. Gut/brain axis and the microbiota. J Clin Invest. 2015; 125:926–38. 10.1172/JCI76304 25689247 PMC4362231

[9] Liang S, Wang T, Hu X, Luo J, Li W, Wu X, Duan Y, Jin F. Administration of Lactobacillus helveticus NS8 improves behavioral, cognitive, and biochemical aberrations caused by chronic restraint stress. Neuroscience. 2015; 310:561–77. 10.1016/j.neuroscience.2015.09.033 26408987

[10] Jiang C, Li G, Huang P, Liu Z, Zhao B. The Gut Microbiota and Alzheimer's Disease. J Alzheimers Dis. 2017; 58:1–15. 10.3233/JAD-161141 28372330

[11] Buford TW. (Dis)Trust your gut: the gut microbiome in age-related inflammation, health, and disease. Microbiome. 2017; 5:80. 10.1186/s40168-017-0296-0 28709450 PMC5512975

[12] Szablewski L. Human Gut Microbiota in Health and Alzheimer’s Disease. J Alzheimers Dis. 2018; 62:549–60. 10.3233/JAD-170908 29480188

[13] Junges VM, Closs VE, Nogueira GM, Gottlieb MG. Crosstalk between Gut Microbiota and Central Nervous System: A Focus on Alzheimer’s Disease. Curr Alzheimer Res. 2018; 15:1179–90. 10.2174/1567205015666180904155908 30182854

[14] Leblhuber F, Egger M, Schuetz B, Fuchs D. Commentary: effect of probiotic supplementation on cognitive function and metabolic status in Alzheimer’s disease: a randomized, double-blind and controlled trial. Front Aging Neurosci. 2018; 10:54. 10.3389/fnagi.2018.00054 29559906 PMC5845584

[15] Wang X, Sun G, Feng T, Zhang J, Huang X, Wang T, Xie Z, Chu X, Yang J, Wang H, Chang S, Gong Y, Ruan L, et al. Sodium oligomannate therapeutically remodels gut microbiota and suppresses gut bacterial amino acids-shaped neuroinflammation to inhibit Alzheimer’s disease progression. Cell Res. 2019; 29:787–803. 10.1038/s41422-019-0216-x 31488882 PMC6796854

[16] Bostanciklioğlu M. The role of gut microbiota in pathogenesis of Alzheimer’s disease. J Appl Microbiol. 2019; 127:954–967. 10.1111/jam.14264 30920075

[17] Vogt NM, Kerby RL, Dill-McFarland KA, Harding SJ, Merluzzi AP, Johnson SC, Carlsson CM, Asthana S, Zetterberg H, Blennow K, Bendlin BB, Rey FE. Gut microbiome alterations in Alzheimer’s disease. Sci Rep. 2017; 7:13537. 10.1038/s41598-017-13601-y 29051531 PMC5648830

[18] Nagpal R, Neth BJ, Wang S, Craft S, Yadav H. Modified Mediterranean-ketogenic diet modulates gut microbiome and short-chain fatty acids in association with Alzheimer’s disease markers in subjects with mild cognitive impairment. EBioMedicine. 2019; 47:529–42. 10.1016/j.ebiom.2019.08.032 31477562 PMC6796564

[19] Kim MS, Kim Y, Choi H, Kim W, Park S, Lee D, Kim DK, Kim HJ, Choi H, Hyun DW, Lee JY, Choi EY, Lee DS, et al. Transfer of a healthy microbiota reduces amyloid and tau pathology in an Alzheimer’s disease animal model. Gut. 2020; 69:283–94. 10.1136/gutjnl-2018-317431 31471351

[20] Kowalski K, Mulak A. Brain-Gut-Microbiota Axis in Alzheimer’s Disease. J Neurogastroenterol Motil. 2019; 25:48–60. 10.5056/jnm18087 30646475 PMC6326209

[21] Cryan JF, O’Riordan KJ, Sandhu K, Peterson V, Dinan TG. The gut microbiome in neurological disorders. Lancet Neurol. 2020; 19:179–194. 10.1016/S1474-4422(19)30356-4 31753762

[22] Lynch SV, Pedersen O. The Human Intestinal Microbiome in Health and Disease. N Engl J Med. 2016; 375:2369–79. 10.1056/NEJMra1600266 27974040

[23] Chunchai T, Thunapong W, Yasom S, Wanchai K, Eaimworawuthikul S, Metzler G, Lungkaphin A, Pongchaidecha A, Sirilun S, Chaiyasut C, Pratchayasakul W, Thiennimitr P, Chattipakorn N, Chattipakorn SC. Decreased microglial activation through gut-brain axis by prebiotics, probiotics, or synbiotics effectively restored cognitive function in obese-insulin resistant rats. J Neuroinflammation. 2018; 15:11. 10.1186/s12974-018-1055-2 29316965 PMC5761137

[24] Wallace CJ, Milev R. The effects of probiotics on depressive symptoms in humans: a systematic review. Ann Gen Psychiatry. 2017; 16:14. 10.1186/s12991-017-0138-2 28239408 PMC5319175

[25] Sharon G, Sampson TR, Geschwind DH, Mazmanian SK. The Central Nervous System and the Gut Microbiome. Cell. 2016; 167:915–32. 10.1016/j.cell.2016.10.027 27814521 PMC5127403

[26] Wang H, Lee IS, Braun C, Enck P. Effect of Probiotics on Central Nervous System Functions in Animals and Humans: A Systematic Review. J Neurogastroenterol Motil. 2016; 22:589–605. 10.5056/jnm16018 27413138 PMC5056568

[27] Akbari E, Asemi Z, Daneshvar Kakhaki R, Bahmani F, Kouchaki E, Tamtaji OR, Hamidi GA, Salami M. Effect of Probiotic Supplementation on Cognitive Function and Metabolic Status in Alzheimer’s Disease: A Randomized, Double-Blind and Controlled Trial. Front Aging Neurosci. 2016; 8:256. 10.3389/fnagi.2016.00256 27891089 PMC5105117

[28] Tamtaji OR, Heidari-Soureshjani R, Mirhosseini N, Kouchaki E, Bahmani F, Aghadavod E, Tajabadi-Ebrahimi M, Asemi Z. Probiotic and selenium co-supplementation, and the effects on clinical, metabolic and genetic status in Alzheimer’s disease: A randomized, double-blind, controlled trial. Clin Nutr. 2019; 38:2569–75. 10.1016/j.clnu.2018.11.034 30642737

[29] Agahi A, Hamidi GA, Daneshvar R, Hamdieh M, Soheili M, Alinaghipour A, Esmaeili Taba SM, Salami M. Does Severity of Alzheimer’s Disease Contribute to Its Responsiveness to Modifying Gut Microbiota? A Double Blind Clinical Trial. Front Neurol. 2018; 9:662. 10.3389/fneur.2018.00662 30158897 PMC6104449

[30] Kobayashi Y, Kuhara T, Oki M, Xiao JZ. Effects of *Bifidobacterium breve* A1 on the cognitive function of older adults with memory complaints: a randomised, double-blind, placebo-controlled trial. Benef Microbes. 2019; 10:511–20. 10.3920/BM2018.0170 31090457

[31] Hwang YH, Park S, Paik JW, Chae SW, Kim DH, Jeong DG, Ha E, Kim M, Hong G, Park SH, Jung SJ, Lee SM, Na KH, et al. Efficacy and Safety of *Lactobacillus Plantarum* C29-Fermented Soybean (DW2009) in Individuals with Mild Cognitive Impairment: A 12-Week, Multi-Center, Randomized, Double-Blind, Placebo-Controlled Clinical Trial. Nutrients. 2019; 11:E305. 10.3390/nu11020305 30717153 PMC6412773

[32] Cheng LH, Liu YW, Wu CC, Wang S, Tsai YC. Psychobiotics in mental health, neurodegenerative and neurodevelopmental disorders. Yao Wu Shi Pin Fen Xi. 2019; 27:632–48. 10.1016/j.jfda.2019.01.002 31324280 PMC9307042

[33] Morris MC, Tangney CC, Wang Y, Sacks FM, Barnes LL, Bennett DA, Aggarwal NT. MIND diet slows cognitive decline with aging. Alzheimers Dement. 2015; 11:1015–22. 10.1016/j.jalz.2015.04.011 26086182 PMC4581900

[34] Alkasir R, Li J, Li X, Jin M, Zhu B. Human gut microbiota: the links with dementia development. Protein Cell. 2017; 8:90–102. 10.1007/s13238-016-0338-6 27866330 PMC5291774

[35] Agostinho P, Cunha RA, Oliveira C. Neuroinflammation, oxidative stress and the pathogenesis of Alzheimer’s disease. Curr Pharm Des. 2010; 16:2766–78. 10.2174/138161210793176572 20698820

[36] Downey LA, Simpson T, Timmer J, Nolidin K, Croft K, Wesnes KA, Scholey A, Deleuil S, Stough C. Impaired verbal episodic memory in healthy older adults is marked by increased F_2_-Isoprostanes. Prostaglandins Leukot Essent Fatty Acids. 2018; 129:32–37. 10.1016/j.plefa.2018.02.001 29482768

[37] Selvarajah D, Wilkinson ID, Davies J, Gandhi R, Tesfaye S. Central nervous system involvement in diabetic neuropathy. Curr Diab Rep. 2011; 11:310–22. 10.1007/s11892-011-0205-z 21667355

[38] Sultana R, Mecocci P, Mangialasche F, Cecchetti R, Baglioni M, Butterfield DA. Increased protein and lipid oxidative damage in mitochondria isolated from lymphocytes from patients with Alzheimer’s disease: insights into the role of oxidative stress in Alzheimer’s disease and initial investigations into a potential biomarker for this dementing disorder. J Alzheimers Dis. 2011; 24:77–84. 10.3233/JAD-2011-101425 21383494

[39] Querfurth HW, LaFerla FM. Alzheimer’s disease. N Engl J Med. 2010; 362:329–44. 10.1056/NEJMra0909142 20107219

[40] Eikelenboom P, van Exel E, Hoozemans JJ, Veerhuis R, Rozemuller AJ, van Gool WA. Neuroinflammation - an early event in both the history and pathogenesis of Alzheimer’s disease. Neurodegener Dis. 2010; 7:38–41. 10.1159/000283480 20160456

[41] Wyss-Coray T. Inflammation in Alzheimer disease: driving force, bystander or beneficial response? Nat Med. 2006; 12:1005–15. 10.1038/nm1484 16960575

[42] McGeer EG, McGeer PL. Neuroinflammation in Alzheimer’s disease and mild cognitive impairment: a field in its infancy. J Alzheimers Dis. 2010; 19:355–61. 10.3233/JAD-2010-1219 20061650

[43] Brown GC, Bal-Price A. Inflammatory neurodegeneration mediated by nitric oxide, glutamate, and mitochondria. Mol Neurobiol. 2003; 27:325–55. 10.1385/MN:27:3:325 12845153

[44] Crouch PJ, Barnham KJ, Duce JA, Blake RE, Masters CL, Trounce IA. Copper-dependent inhibition of cytochrome c oxidase by Abeta(1-42) requires reduced methionine at residue 35 of the Abeta peptide. J Neurochem. 2006; 99:226–36. 10.1111/j.1471-4159.2006.04050.x 16987248

[45] Manczak M, Anekonda TS, Henson E, Park BS, Quinn J, Reddy PH. Mitochondria are a direct site of A beta accumulation in Alzheimer’s disease neurons: implications for free radical generation and oxidative damage in disease progression. Hum Mol Genet. 2006; 15:1437–49. 10.1093/hmg/ddl066 16551656

[46] Wilkinson BL, Landreth GE. The microglial NADPH oxidase complex as a source of oxidative stress in Alzheimer’s disease. J Neuroinflammation. 2006; 3:30. 10.1186/1742-2094-3-30 17094809 PMC1637099

[47] Matos M, Augusto E, Oliveira CR, Agostinho P. Amyloid-beta peptide decreases glutamate uptake in cultured astrocytes: involvement of oxidative stress and mitogen-activated protein kinase cascades. Neuroscience. 2008; 156:898–910. 10.1016/j.neuroscience.2008.08.022 18790019

[48] Clark A, Mach N. The Crosstalk between the Gut Microbiota and Mitochondria during Exercise. Front Physiol. 2017; 8:319. 10.3389/fphys.2017.00319 28579962 PMC5437217

[49] D’Souza A, Fordjour L, Ahmad A, Cai C, Kumar D, Valencia G, Aranda JV, Beharry KD. Effects of probiotics, prebiotics, and synbiotics on messenger RNA expression of caveolin-1, NOS, and genes regulating oxidative stress in the terminal ileum of formula-fed neonatal rats. Pediatr Res. 2010; 67:526–31. 10.1203/PDR.0b013e3181d4ff2b 20101198

[50] Athari Nik Azm S, Djazayeri A, Safa M, Azami K, Ahmadvand B, Sabbaghziarani F, Sharifzadeh M, Vafa M. Lactobacilli and bifidobacteria ameliorate memory and learning deficits and oxidative stress in β-amyloid (1-42) injected rats. Appl Physiol Nutr Metab. 2018; 43:718–26. 10.1139/apnm-2017-0648 29462572

[51] Kobayashi Y, Sugahara H, Shimada K, Mitsuyama E, Kuhara T, Yasuoka A, Kondo T, Abe K, Xiao JZ. Therapeutic potential of Bifidobacterium breve strain A1 for preventing cognitive impairment in Alzheimer’s disease. Sci Rep. 2017; 7:13510. 10.1038/s41598-017-13368-2 29044140 PMC5647431

[52] Nimgampalle M, Kuna Y. Anti-Alzheimer Properties of Probiotic, *Lactobacillus plantarum* MTCC 1325 in Alzheimer’s Disease induced Albino Rats. J Clin Diagn Res. 2017; 11:KC01–05. 10.7860/JCDR/2017/26106.10428 28969160 PMC5620801

[53] Musa NH, Mani V, Lim SM, Vidyadaran S, Abdul Majeed AB, Ramasamy K. Lactobacilli-fermented cow’s milk attenuated lipopolysaccharide-induced neuroinflammation and memory impairment in vitro and in vivo. J Dairy Res. 2017; 84:488–95. 10.1017/S0022029917000620 29154736

[54] Gibson GR, Hutkins R, Sanders ME, Prescott SL, Reimer RA, Salminen SJ, Scott K, Stanton C, Swanson KS, Cani PD, Verbeke K, Reid G. Expert consensus document: the International Scientific Association for Probiotics and Prebiotics (ISAPP) consensus statement on the definition and scope of prebiotics. Nat Rev Gastroenterol Hepatol. 2017; 14:491–502. 10.1038/nrgastro.2017.75 28611480

[55] FAO/WHO. Guidelines for the evaluation of probiotics in food. Report of a Joint FAO/WHO working group on drafting guidelines for the evaluation of probioticsin food. FAO/WHO; 2002.

[56] Hill C, Guarner F, Reid G, Gibson GR, Merenstein DJ, Pot B, Morelli L, Canani RB, Flint HJ, Salminen S, Calder PC, Sanders ME. Expert consensus document. The International Scientific Association for Probiotics and Prebiotics consensus statement on the scope and appropriate use of the term probiotic. Nat Rev Gastroenterol Hepatol. 2014; 11:506–14. 10.1038/nrgastro.2014.66 24912386

[57] McFarland LV. Meta-analysis of probiotics for the prevention of antibiotic associated diarrhea and the treatment of Clostridium difficile disease. Am J Gastroenterol. 2006; 101:812–22. 10.1111/j.1572-0241.2006.00465.x 16635227

[58] Van Niel CW, Feudtner C, Garrison MM, Christakis DA. Lactobacillus therapy for acute infectious diarrhea in children: a meta-analysis. Pediatrics. 2002; 109:678–84. 10.1542/peds.109.4.678 11927715

[59] Johnston BC, Goldenberg JZ, Vandvik PO, Sun X, Guyatt GH. Probiotics for the prevention of pediatric antibiotic-associated diarrhea. Cochrane Database Syst Rev. 2011; CD004827. 10.1002/14651858.CD004827.pub3 22071814

[60] Goldenberg JZ, Ma SS, Saxton JD, Martzen MR, Vandvik PO, Thorlund K, Guyatt GH, Johnston BC. Probiotics for the prevention of Clostridium difficile-associated diarrhea in adults and children. Cochrane Database Syst Rev. 2013; CD006095. 10.1002/14651858.CD006095.pub3 23728658

[61] McFarland LV. Probiotics for the Primary and Secondary Prevention of C. difficile Infections: A Meta-analysis and Systematic Review. Antibiotics (Basel). 2015; 4:160–78. 10.3390/antibiotics4020160 27025619 PMC4790329

[62] Lau CS, Chamberlain RS. Probiotics are effective at preventing Clostridium difficile-associated diarrhea: a systematic review and meta-analysis. Int J Gen Med. 2016; 9:27–37. 10.2147/IJGM.S98280 26955289 PMC4769010

[63] Aceti A, Gori D, Barone G, Callegari ML, Di Mauro A, Fantini MP, Indrio F, Maggio L, Meneghin F, Morelli L, Zuccotti G, Corvaglia L, and Italian Society of Neonatology. Probiotics for prevention of necrotizing enterocolitis in preterm infants: systematic review and meta-analysis. Ital J Pediatr. 2015; 41:89. 10.1186/s13052-015-0199-2 26567539 PMC4644279

[64] Pelucchi C, Chatenoud L, Turati F, Galeone C, Moja L, Bach JF, La Vecchia C. Probiotics supplementation during pregnancy or infancy for the prevention of atopic dermatitis: a meta-analysis. Epidemiology. 2012; 23:402–14. 10.1097/EDE.0b013e31824d5da2 22441545

[65] Zhang GQ, Hu HJ, Liu CY, Zhang Q, Shakya S, Li ZY. Probiotics for Prevention of Atopy and Food Hypersensitivity in Early Childhood: A PRISMA-Compliant Systematic Review and Meta-Analysis of Randomized Controlled Trials. Medicine (Baltimore). 2016; 95:e2562. 10.1097/MD.0000000000002562 26937896 PMC4778993

[66] Miller LE, Zimmermann AK, Ouwehand AC. Contemporary meta-analysis of short-term probiotic consumption on gastrointestinal transit. World J Gastroenterol. 2016; 22:5122–31. 10.3748/wjg.v22.i21.5122 27275105 PMC4886388

[67] Ouwehand AC. A review of dose-responses of probiotics in human studies. Benef Microbes. 2017; 8:143–51. 10.3920/BM2016.0140 28008787

[68] Sanders ME, Merenstein D, Merrifield CA, Hutkins R. Hutkins. Probiotics for human use. Nutr Bull. 2018; 43:212–25. 10.1111/nbu.12334

[69] Larsen CN, Nielsen S, Kaestel P, Brockmann E, Bennedsen M, Christensen HR, Eskesen DC, Jacobsen BL, Michaelsen KF. Dose-response study of probiotic bacteria Bifidobacterium animalis subsp lactis BB-12 and Lactobacillus paracasei subsp paracasei CRL-341 in healthy young adults. Eur J Clin Nutr. 2006; 60:1284–93. 10.1038/sj.ejcn.1602450 16721394

[70] Hibberd PL, Kleimola L, Fiorino AM, Botelho C, Haverkamp M, Andreyeva I, Poutsiaka D, Fraser C, Solano-Aguilar G, Snydman DR. No evidence of harms of probiotic Lactobacillus rhamnosus GG ATCC 53103 in healthy elderly-a phase I open label study to assess safety, tolerability and cytokine responses. PLoS One. 2014; 9:e113456. 10.1371/journal.pone.0113456 25438151 PMC4249962

[71] van de Zande E, van de Nes JC, Jansen I, van den Berg MN, Zwart AF, Bimmel D, Rijkers GT, Andringa G. The Test Your Memory (TYM) Test Outperforms the MMSE in the Detection of MCI and Dementia. Curr Alzheimer Res. 2017; 14:598–607. 10.2174/1567205013666161201202520 28000550

[72] Panza F, Solfrizzi V, Imbimbo BP, Tortelli R, Santamato A, Logroscino G. Amyloid-based immunotherapy for Alzheimer’s disease in the time of prevention trials: the way forward. Expert Rev Clin Immunol. 2014; 10:405–19. 10.1586/1744666X.2014.883921 24490853

[73] Panza F, Solfrizzi V, Seripa D, Imbimbo BP, Lozupone M, Santamato A, Zecca C, Barulli MR, Bellomo A, Pilotto A, Daniele A, Greco A, Logroscino G. Tau-Centric Targets and Drugs in Clinical Development for the Treatment of Alzheimer’s Disease. BioMed Res Int. 2016; 2016:3245935. 10.1155/2016/3245935 27429978 PMC4939203

[74] Gautam RRG. Pipeline assessment of beta-secretase inhibitors for alzheimer’s disease: hopes or gloom for a trillion dollar market. Value Health. 2017; 20:A887 10.1016/j.jval.2017.08.2654

[75] Dinan TG, Cryan JF. The Microbiome-Gut-Brain Axis in Health and Disease. Gastroenterol Clin North Am. 2017; 46:77–89. 10.1016/j.gtc.2016.09.007 28164854

[76] Angelucci F, Cechova K, Amlerova J, Hort J. Antibiotics, gut microbiota, and Alzheimer’s disease. J Neuroinflammation. 2019; 16:108. 10.1186/s12974-019-1494-4 31118068 PMC6530014

[77] Higgins J, Green S. Cochrane Handbook for Systematic Reviews of Interventions, Version 5.1.0. 2013.

