# Supplementary material for: Efficacy of probiotics on cognition, and biomarkers of inflammation and oxidative stress in adults with Alzheimer’s disease or mild cognitive impairment — a meta-analysis of randomized controlled trials
Source: Aging (Albany NY). 2020 Feb 15;12(4):4010–39. doi: 10.18632/aging.102810 (PMC7066922; doi:10.18632/aging.102810)
Supplement: Supplementary Materials [file aging-12-102810-s002..pdf]

SUPPLEMENTARY MATERIALS

Details of searching strategy and screening process

Literature search

The specific searching strategy and results for each database were described as follows:

EMBASE

Search strategy: ('alzheimer disease'/exp OR 'alzeimer disease' OR 'alzeimer`s disease' OR 'alzeimers disease' OR 'alzheimer dementia' OR 'alzheimer disease' OR 'alzheimers disease' OR 'alzheimer fibrillary change' OR 'alzheimer fibrillary lesion'OR 'alzheimer neurofibrillary change' OR 'alzheimer neurofibrillary degeneration' OR 'alzheimer neuron degeneration' OR 'alzheimer perusini disease' OR 'alzheimer sclerosis' OR 'alzheimer syndrome' OR 'alzheimer`s disease' OR 'cortical sclerosis, diffuse' OR 'dementia, alzheimer' OR 'diffuse cortical sclerosis' OR 'late onset alzheimer disease' OR 'dementia'/exp OR 'amentia' OR 'dementia' OR 'demention' OR 'mild cognitive impairment'/exp OR 'amnestic mild cognitive impairment' OR 'mild cognitive impairment' OR 'cognitive defect'/exp OR 'cognition disorder' OR 'cognition disorders' OR 'cognitive defect' OR 'cognitive defects' OR 'cognitive deficit' OR 'cognitive disability' OR 'cognitive disorder' OR 'cognitive disorders' OR 'cognitive impairment' OR 'delirium, dementia, amnestic, cognitive disorders' OR 'overinclusion' OR 'response interference' OR 'cognition'/exp OR 'cognition' OR 'cognitive accessibility'OR 'cognitive balance' OR 'cognitive dissonance' OR 'cognitive function' OR 'cognitive structure' OR 'cognitive symptoms' OR 'cognitive task' OR 'cognitive thinking' OR 'neurobehavioural manifestations' OR 'volition' OR 'memory'/exp OR 'item recall' OR 'memory' OR 'memory function' OR 'nonspatial memory' OR 'remembering' OR 'reminiscence' OR 'mental capacity'/exp OR 'ability, mental' OR 'attainment' OR 'capacity, mental' OR 'fitness, mental' OR 'mental ability' OR 'mental capacity' OR 'mental competency' OR ad OR mci OR 'cognitive fail*' OR 'cognitive decline*' OR 'cognitive impair*' OR alzheimer* OR dement* OR 'cognitive dysfunction' OR 'cognitive performance' OR cognitive) AND ('probiotic agent'/exp OR 'probiotic' OR 'probiotic agent' OR 'probiotics' OR 'yeast'/exp OR 'flora, yeast' OR 'fungi, yeast' OR 'yeast' OR 'yeast fermentation' OR 'yeast flora' OR 'yeast fungus' OR 'yeast germination' OR 'yeast metabolism' OR 'yeasts' OR 'yoghurt'/exp OR 'yoghourt' OR 'yoghurt' OR 'yogurt' OR 'zabadi' OR 'lactobacillus'/exp OR 'betabacterium' OR 'lactobacileae' OR 'lactobacilleae' OR 'lactobacillus' OR 'lactobacteria' OR 'lactobacilli' OR 'bifidobacterium'/exp OR 'bifidobacterium' OR 'fermented dairy product'/exp OR 'cultured dairy foods' OR 'cultured dairy product' OR 'cultured milk foods' OR 'cultured milk product' OR 'cultured milk products' OR 'fermented dairy foods' OR 'fermented dairy product' OR 'fermented milk' OR 'fermented milk product' OR 'fermented product'/exp OR 'fermented food' OR 'fermented foods' OR 'fermented product' OR 'synbiotic agent'/exp OR 'synbiotic' OR 'synbiotic agent' OR 'synbiotics' OR probiotic*) AND ('placebo'/exp OR 'placebo' OR 'placebo gel' OR 'placebos') AND ('randomized controlled trial'/exp OR 'controlled trial, randomized' OR 'randomised controlled study' OR 'randomised controlled trial' OR 'randomized controlled study' OR 'randomized controlled trial' OR 'trial, randomized controlled' OR 'controlled clinical trial'/exp OR 'clinical trial, controlled' OR 'controlled clinical comparison' OR 'controlled clinical drug trial' OR 'controlled clinical experiment' OR 'controlled clinical study' OR 'controlled clinical test' OR 'controlled clinical trial' OR rct OR random* OR control* OR trial*) AND 'human'/de AND ([adult]/lim OR [aged]/lim OR [middle aged]/lim OR [very elderly]/lim OR [young adult]/lim)

Search results: 149 items

PubMed

Search strategy: (((("Adult"[Mesh]) AND "Humans"[Mesh]) AND (((((((((((((((((("Alzheimer Disease"[Mesh]) OR Alzheimer*) OR AD) OR "Dementia"[Mesh]) OR Dement*) OR mild cognitive impairment) OR MCI) OR ("Cognition"[Mesh] OR "Cognition Disorders"[Mesh])) OR ("Memory"[Mesh] OR "Memory Disorders"[Mesh])) OR "Cognitive Dysfunction"[Mesh]) OR cognitive defect) OR Mental capacity) OR Cognitive) OR cognitive performance) OR cognitive impair*) OR cognitive decline*) OR cognitive fail*)) AND ("Placebos"[Mesh]) AND ((((((((((" Probiotics"[Mesh]) OR "Cultured Milk Products"[Mesh]) OR "Yogurt"[Mesh]) OR (("Yeast, Dried"[Mesh]) OR "Yeasts"[Mesh])) OR "Bifidobacterium"[Mesh]) OR "Lactobacillus"[Mesh]) OR "Synbiotics"[Mesh]) OR probiotic*) OR fermented product) OR fermented dairy product)) AND ((((((((("Randomized Controlled Trial"[Publication Type] OR "Controlled Clinical Trial"[Publication Type])) OR randomized controlled trials) OR Controlled Clinical Trials) OR RCT) OR random*) OR control*) OR trial*)))))

Search results: 111 items

Cochrane Central Register of Controlled Trials (CENTRAL)

Search strategy:

ID	Search	Hits
#1	MeSH descriptor: [Lactobacillus] explode all trees	1487
#2	MeSH descriptor: [Yogurt] explode all trees	329
#3	MeSH descriptor: [Probiotics] explode all trees	1796
#4	MeSH descriptor: [Bifidobacterium] explode all trees	655
#5	MeSH descriptor: [Yeasts] explode all trees	711
#6	MeSH descriptor: [Cultured Milk Products] explode all trees	499
#7	MeSH descriptor: [Synbiotics] explode all trees	118
#8	#1 or #2 or #3 or #4 or #5 or #6 or #7	3356
#9	"probiotic*" or "fermented product" or "fermented dairy product"	4357
#10	#8 or #9	6286
#11	adult and humans	351364
#12	MeSH descriptor: [Alzheimer Disease] explode all trees	3015
#13	MeSH descriptor: [Dementia] explode all trees	5224
#14	MeSH descriptor: [Cognitive Dysfunction] explode all trees	922
#15	MeSH descriptor: [Cognition] explode all trees	9641
#16	MeSH descriptor: [Memory] explode all trees	7470
#17	MeSH descriptor: [Cognition Disorders] explode all trees	4475
#18	MeSH descriptor: [Memory Disorders] explode all trees	1148
#19	Alzheimer* or AD or Dement* or "cognitive" or "mild cognitive impairment" or MCI or "cognitive fail*" or "cognitive performance" or "cognitive impair*" or "cognitive decline*" or "memory function" or "Mental capacity" or "cognitive defect"	99308
#20	#12 or #13 or #14 or #15 or #16 or #17 or #18 or #19	107156
#21	MeSH descriptor: [Randomized Controlled Trial] explode all trees	124
#22	MeSH descriptor: [Controlled Clinical Trial] explode all trees	132
#23	RCT or "randomized controlled trials" or "Controlled Clinical Trials" or "random*" or "control*" or "trial*"	1159526
#24	#21 or #22 or #23	1159526
#25	#10 and #20 and #24	302
#26	#25 and trails	282

Search results: 282 items

Web of Science

Search strategy: ('alzheimer disease'/exp OR 'alzeimer disease' OR 'alzeimer`s disease' OR 'alzeimers disease' OR 'alzheimer dementia' OR 'alzheimer disease' OR 'alzheimers disease' OR 'alzheimer fibrillary change' OR 'alzheimer fibrillary lesion'OR 'alzheimer neurofibrillary change' OR 'alzheimer neurofibrillary degeneration' OR 'alzheimer neuron degeneration' OR 'alzheimer perusini disease' OR 'alzheimer sclerosis' OR 'alzheimer syndrome' OR 'alzheimer`s disease' OR 'cortical sclerosis, diffuse' OR 'dementia, alzheimer' OR 'diffuse cortical sclerosis' OR 'late onset alzheimer disease' OR 'dementia'/exp OR 'amentia' OR 'dementia' OR 'demention' OR 'mild cognitive impairment'/exp OR 'amnestic mild cognitive impairment' OR 'mild cognitive impairment' OR 'cognitive defect'/exp OR 'cognition disorder' OR 'cognition disorders' OR 'cognitive defect' OR 'cognitive defects' OR 'cognitive deficit' OR 'cognitive disability' OR 'cognitive disorder' OR 'cognitive disorders' OR 'cognitive impairment' OR 'delirium, dementia, amnestic, cognitive disorders' OR 'overinclusion' OR 'response interference' OR 'cognition'/exp OR 'cognition' OR 'cognitive accessibility'OR 'cognitive balance' OR 'cognitive dissonance' OR 'cognitive function' OR 'cognitive structure' OR 'cognitive symptoms' OR 'cognitive task' OR 'cognitive thinking' OR 'neurobehavioural manifestations' OR 'volition' OR 'memory'/exp OR 'item recall' OR 'memory' OR 'memory function' OR 'nonspatial memory' OR 'remembering' OR 'reminiscence' OR 'mental capacity'/exp OR 'ability, mental' OR 'attainment' OR 'capacity, mental' OR 'fitness, mental' OR 'mental ability' OR 'mental capacity' OR 'mental competency' OR ad OR mci OR 'cognitive fail*' OR 'cognitive decline*' OR 'cognitive impair*' OR alzheimer* OR dement* OR 'cognitive dysfunction' OR 'cognitive performance' OR cognitive) AND ('probiotic agent'/exp OR 'probiotic' OR 'probiotic agent' OR 'probiotics' OR 'yeast'/exp OR 'flora, yeast' OR 'fungi, yeast' OR 'yeast' OR 'yeast fermentation' OR 'yeast flora' OR 'yeast fungus' OR 'yeast germination' OR 'yeast metabolism' OR 'yeasts' OR 'yoghurt'/exp OR 'yoghourt' OR 'yoghurt' OR 'yogurt' OR 'zabadi' OR 'lactobacillus'/exp OR 'betabacterium' OR 'lactobacileae' OR 'lactobacilleae' OR 'lactobacillus' OR 'lactobacteria' OR 'lactobacilli' OR

'bifidobacterium'/exp OR 'bifidobacterium' OR 'fermented dairy product'/exp OR 'cultured dairy foods' OR 'cultured dairy product' OR 'cultured milk foods' OR 'cultured milk product' OR 'cultured milk products' OR 'fermented dairy foods' OR 'fermented dairy product' OR 'fermented milk' OR 'fermented milk product' OR 'fermented product'/exp OR 'fermented food' OR 'fermented foods' OR 'fermented product' OR 'synbiotic agent'/exp OR 'synbiotic' OR 'synbiotic agent' OR 'synbiotics' OR probiotic*) AND ('randomized controlled trial'/exp OR 'controlled trial, randomized' OR 'randomised controlled study' OR 'randomised controlled trial' OR 'randomized controlled study' OR 'randomized controlled trial' OR 'trial, randomized controlled' OR 'controlled clinical trial'/exp OR 'clinical trial, controlled' OR 'controlled clinical comparison' OR 'controlled clinical drug trial' OR 'controlled clinical experiment' OR 'controlled clinical study' OR 'controlled clinical test' OR 'controlled clinical trial' OR rct OR random* OR control* OR trial*) AND (Human AND adult)

Search results: 341 items

Literature screening

All studies were imported to Endnote X6. After discarding the duplicates, 687studies were remained. The title and abstract screening excluded 667 papers with 20 papers remained. The full-text screening further excluded 15 studies, with the remaining 5 studies included in the meta-analysis. The 15 studies excluded according reasons as follows:

No.	Author	Year	Title	Source	Decision	Notes
1	Irct2015113056 23N.	2015	Effect of supplementation in treatment of patients with Alzheimer's disease.	https://www.cochranelibrary.com/central/doi/10.1002/central/CN-01814566/full	Exclude: register information of included research (Akbari, 2016)	IRCT20151130 5623N60
2	Irct2017061534 549N.	2017	The effect of probiotic supplementation on cognitive function and inflammatory markers in patients with early and late phases of Alzheimer's disease.	https://www.cochranelibrary.com/central/doi/10.1002/central/CN-01892678/full	Exclude: register information of included research (Aghi.A, 2018)	IRCT20170615 34549N1
3	Irct2017061203 4497N.	2018	Effect of combined probiotic and selenium supplementation in treatment of patients with Alzheimer's disease.	https://www.cochranelibrary.com/central/doi/10.1002/central/CN-01896860/full	Exclude: register information of included research (Omid Reza Tamtaji, 2018)	IRCT20170612 034497N5
4	Jicha, G. A.	2015	A phase I, randomized, double-blind, placebo-controlled, multiple ascending dose study of AT-001 yeast selenium for the prevention of Alzheimer's disease.	Alzheimer's and Dementia, 11(7), P471.	Exclude: Conference abstract	Alzheimer's Association International Conference 2015
5	Jung Park, H.	2019	A randomized, double-blind, placebo-controlled study on the memory-enhancing effect of lactobacillus fermented Saccharina japonica extract.	European Journal of Integrative Medicine, 28, 39-46. doi: 10.1016/j.eujim.2019.04.006	Exclude: Inclusion criteria for participants not met	
6	Kim KY	2018	Association between diets and mild cognitive impairment in adults aged 50 years or older.	Nutrition Research and Practice, 12(5), 415-425. doi: 10.4162/nrp.2018.12.5.415	Exclude: not an RCT	

7	Louzada, E. R.	2018	Synbiotic supplementation, systemic inflammation, and symptoms of brain disorders in elders: A secondary study from a randomized clinical trial.	Nutritional Neuroscience, 1-8. doi: 10.1080/1028415X.2018.1477349	Exclude: Inclusion criteria for participants not met	
8	Nct.	2013	Cognitive and Metabolic Effects of a Probiotic Supplement.	https://clinicaltrials.gov/ct2/show/nct02005003	Exclude: Inclusion criteria for participants not met	Accepts Healthy Volunteers: Yes; Healthy (self-reported) and not on medication
9	Nct.	2017	Probiotic on Psychological and Cognitive Effects.	https://clinicaltrials.gov/show/nct03080818.	Exclude: Inclusion criteria for participants not met	Accepts Healthy Volunteers: Yes
10	Nct.	2018	Examining the Effects of One-Month Probiotic Treatment on Mental Fatigue.	https://clinicaltrials.gov/show/nct03611478.	Exclude: Inclusion criteria for participants not met	The aim of this study is to demonstrate the effects of 28-days supplementation with a novel probiotic formulation on mental fatigue following a cognitive load in healthy adults
11	Nct.	2018	Understanding gut feelings: probiotics and cognition.	https://www.cochranelibrary.com/central/doi/10.1002/central/CN-01906153/full	Exclude: Inclusion criteria for participants not met	Inclusion criteria: Healthy adult participants
12	Owen, L.	2014	A double blind, placebo controlled, randomised pilot trial examining the effects of probiotic administration on mood and cognitive function.	Proceedings of the Nutrition Society, 73(OCE1), E29-E29. doi: 10.1017/s0029665114000433	Exclude: Conference abstract	Winter Meeting, 11–12 December 2013, Diet, gut microbiology and human health
13	Pelka, R. B.	1995	Pre-Alzheimer study. Effect of a plant yeast extract (Bio-Strath®) in a randomized double-blind study.	Ars Medici, 85(1), 54-61.	Exclude: No full text form	
14	Friedrich Leblhuber	2018	Probiotic Supplementation in Patients with Alzheimer's Dementia - An Explorative Intervention Study	Current Alzheimer Research, 2018, 15, 1106-1113	Exclude: no cognition assessment	
15	M. CARLSSON	2009	The feasibility of serving liquid yoghurt supplemented with probiotic bacteria, Lactobacillus rhamnosus LB 21, and Lactococcus lactis L1A - A pilot study among old people with dementia in a residential care facility	The Journal of Nutrition, Health & Aging©Volume 13, Number 9, 2009	Exclude: no cognition assessment	

Detailed results of subgroup analyses, publication bias and sensitivity analysis

Subgroup analyses

Subgroup by type of diseases:

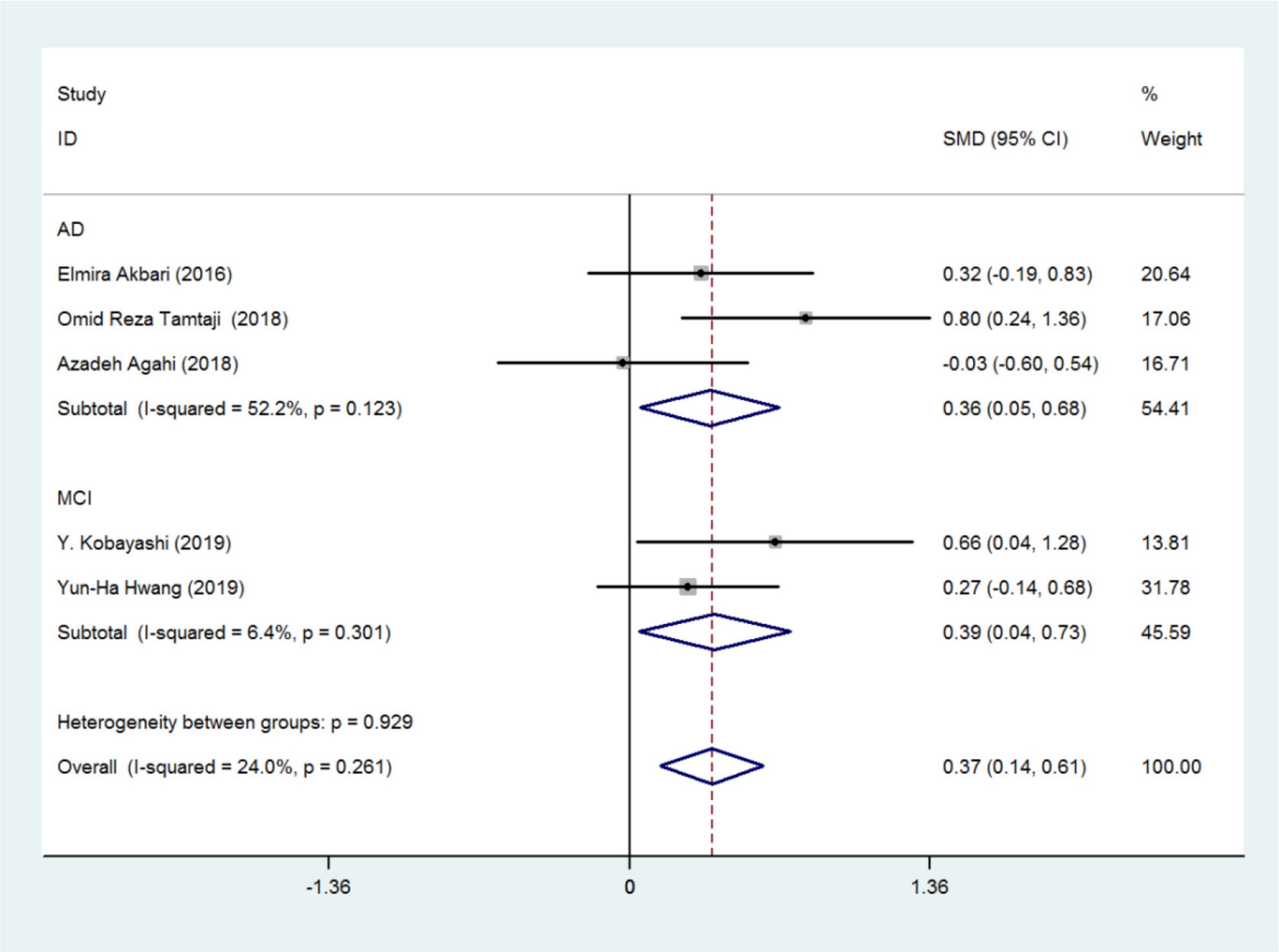
Study
ID
SMD (95% CI)
%
Weight
AD
Elmira Akbari (2016)
0.32 (-0.19, 0.83)
20.64
Omid Reza Tamtaji (2018)
0.80 (0.24, 1.36)
17.06
Azadeh Agahi (2018)
-0.03 (-0.60, 0.54)
16.71
Subtotal (I-squared = 52.2%, p = 0.123)
0.36 (0.05, 0.68)
54.41
MCI
Y. Kobayashi (2019)
0.66 (0.04, 1.28)
13.81
Yun-Ha Hwang (2019)
0.27 (-0.14, 0.68)
31.78
Subtotal (I-squared = 6.4%, p = 0.301)
0.39 (0.04, 0.73)
45.59
Heterogeneity between groups: p = 0.929
Overall (I-squared = 24.0%, p = 0.261)
0.37 (0.14, 0.61)
100.00
-1.36
0
1.36

Subgroup by the cognitive rating scales:

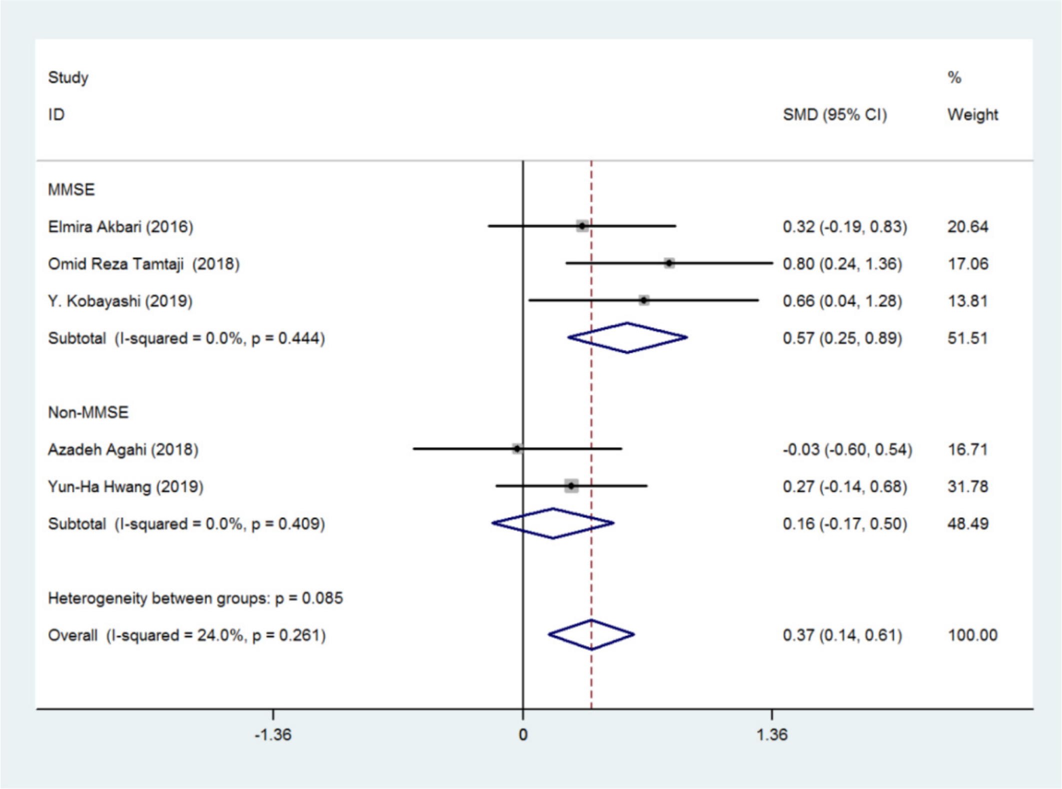

Subgroup by the strains of flora:

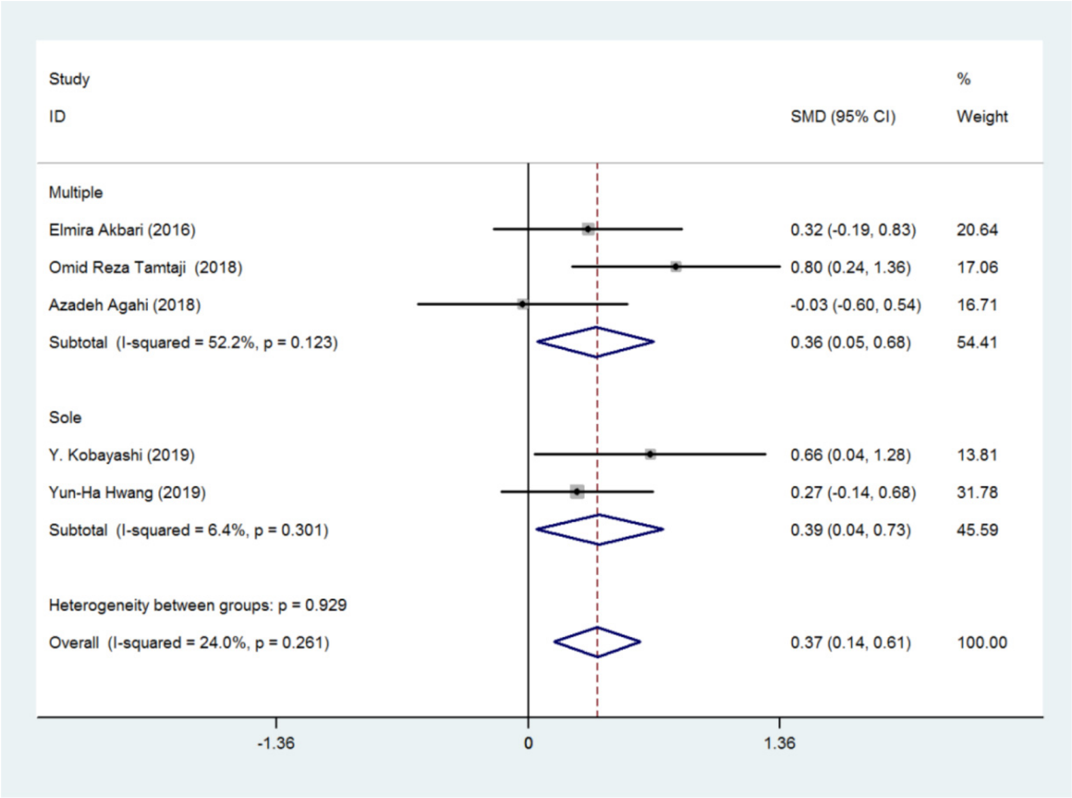

Publication bias

Begg's funnel plot and test

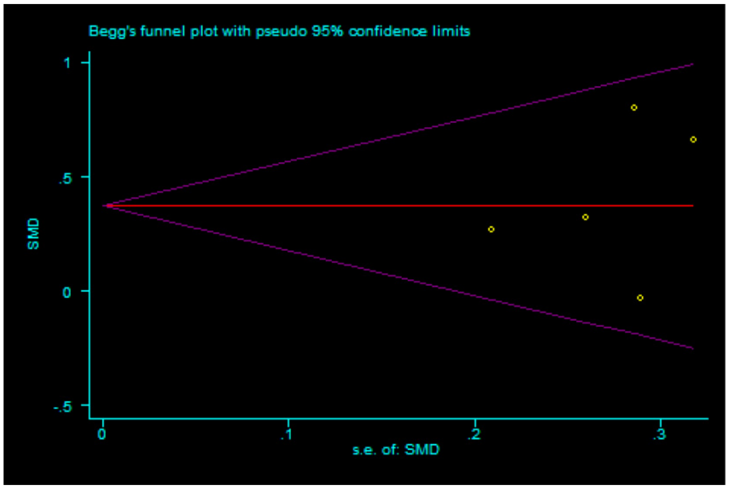

adj. Kendall's Score (P-Q) = 2
Std. Dev. of Score = 4.08
Number of Studies = 5
z = 0.49
Pr > |z| = 0.624
z = 0.24 (continuity corrected)
Pr > |z| = 0.806 (continuity corrected)

Egger's test

Std_Eff	Coef.	Std. Err.	t	P>|t|	[95% Conf. Interval]	
slope	-.3018107	.9814531	-0.31	0.779	-3.425232	2.821611
bias	2.586162	3.716345	0.70	0.537	-9.240906	14.41323

Sensitivity analysis (Leave-one-out influence analysis)

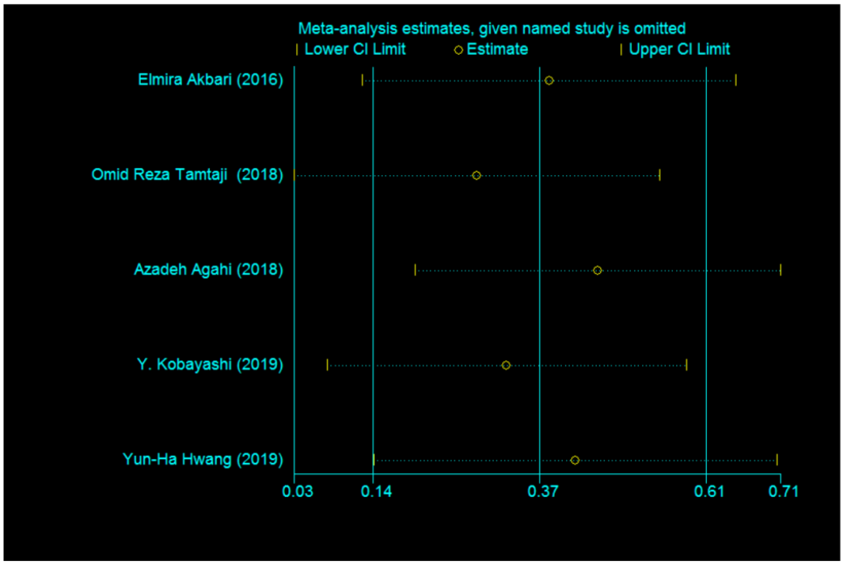

Study omitted |	Estimate	[95% Conf. Interval]	
Elmira Akbari (2016)|	.3868553	.1270016	.64670902
Omid Reza Tamtaji (2018)|	.28621155	.03203233	.54039079
Azadeh Agahi (2018)|	.45443958	.2008017	.70807743
Y. Kobayashi (2019)|	.3278729	.07854173	.57720405
Yun-Ha Hwang (2019)|	.42384291	.14358303	.70410275
Combined |	.37365879	.14217712	.60514046

A Research Protocol to Guide the Meta-Analysis of Efficacy of Probiotics in Alzheimer's Disease or Mild Cognitive Impairment Patients Studies

INTRODUCTION

Rationale

The incidence of Alzheimer's disease (AD) is increasing globally and has reached the point of being a costly public health issue [1]. Mild cognitive impairment (MCI) is a syndrome defined as cognitive deterioration that does not compromise daily functioning; however, amnestic MCI has a high risk of progression to AD and has been considered as a typical prodromal stage of AD [2]. Unfortunately, there is currently no curative treatment for cognitive impairment and dementia [3].

The gut microbiota (GM) consists of a vast bacterial community that resides primarily in the lower gut and lives in a symbiotic relationship with the host [4]. Accumulating evidence has demonstrated the close interconnection between the gastrointestinal tract and the brain, known as the gut-brain axis [5]. Recently, the GM has been found to regulate brain development and behavior via the gut-brain axis, and this has been called the microbiota-gut-brain axis (MGB) [6]. It has been found that dysfunction in behavior and cognition are associated with GM dysbiosis [7], and further activation of gut inflammation has been regarded as a possible pathogenic cofactor in cognitive deterioration and dementia [8]. Moreover, decreased abundance of anti-inflammatory bacterial species such as *Bifidobacterium breve* strain A1 and increased abundance of pro-inflammatory flora phyla such as *Firmicutes* and *Bacteroidetes* are the most distinctive alterations in the GM observed in AD patients [9]. Therefore, the GM has been proposed as a key player in the pathogenesis of AD and might be a new potential therapeutic target for the prevention and treatment of AD [10].

Probiotics are live microbes that confer health benefits to the host when administered in adequate amounts [11], possibly through their anti-inflammatory or anti-oxidative effects [12, 13]. Recently, some probiotics have been shown to influence the central nervous system (CNS) and behavior via modulation of the MGB [14]. Moreover, eleven preclinical studies have shown that neither single strains nor multi-strain probiotics were beneficial for improving cognitive function in animal models [15]. These preclinical results have indicated that probiotics supplementation might be an effective dietary intervention to ameliorate age-associated cognitive deficits. Nevertheless, findings from available clinical trials focusing on the effects of probiotics in patients with AD or MCI are inconsistent [16–20]. Additionally, previous relevant reviews mainly focused on the effect of probiotics in neurodegenerative and neurodevelopmental disorders with both animal models and human trials [15, 21]. The evidence for the effects of psychobiotics on mental and neurological conditions/disorders remains limited. Thus, a meta-analysis is needed to clarify the efficacy of probiotics on cognition in adults suffering from AD or MCI, as well as the possible underlying mechanisms. Given the potential importance of such a meta-analysis, this protocol provides an in-depth description of the research objectives as well as the methodological and analytical approaches that will be used to identify, appraise, and synthesize the relevant studies.

Objectives

(1) Conduct a meta-analysis to clarify the efficacy of probiotics on cognition in adults suffering from AD or MCI.
(2) To examine the effect of probiotics on inflammatory and oxidative biomarkers in adults with AD or MCI.

METHODS

Study selection

Eligibility criteria
(1) Adult human participants who had a diagnosis of AD or MCI (aged over 18 y);
(2) The study was an RCT and published in peer-reviewed journals in English;
(3) No constraint on dosage, strain, or form of probiotics (either stand alone or in combination with

other compounds) was applied;
(4) Probiotic interventions had no significant difference in form, appearance, taste, and smell compared to the control interventions;
(5) No restrictions were applied on validated measures of cognitive assessment;
(6) Continuous data at baseline and post-intervention, or the change from baseline, were reported or could be calculated from the data reported in the article.

Exclusion criteria

(1) The publications were abstracts, reviews, conference papers, study protocols, cross-sectional studies, nonhuman (in vitro and animal) studies or papers that did not report on any outcome of interest;

(2) No post-intervention or change from baseline on scale scores of cognition was reported, and these data could not be calculated based on the information in the article; (3) The study reported on a sample that overlapped the sample in another study. In this case, only the study with the larger sample size was included.

Information sources

The preliminary search will be performed through electronic databases including the EMBASE, PubMed, Web of Science and the Cochrane Central Register of Controlled Trials (CENTRAL). And a snowball search will be manually carried out by searching reference lists from relevant published reviews and the retrieved papers.

Search strategy

The search strategy was developed, finalized and adapted for each database with a combination of free text and controlled vocabulary keywords, including the following terms: (Alzheimer’s disease OR dementia OR mild cognitive impairment OR cognitive dysfunction OR cognitive defect OR cognition OR memory OR mental capacity) AND (adult OR human) AND (probiotic OR yeast OR yoghurt OR fermented product OR lactobacillus OR bifidobacterium OR fermented dairy product OR synbiotics OR cultured milk products). Predefined search strategy for each electronic databases are as follows:

EMBASE

Search strategy: ('alzheimer disease'/exp OR 'alzeimer disease' OR 'alzeimer`s disease' OR 'alzeimers disease' OR 'alzheimer dementia' OR 'alzheimer disease' OR 'alzheimers disease' OR 'alzheimer fibrillary change' OR 'alzheimer fibrillary lesion'OR 'alzheimer neurofibrillary change' OR 'alzheimer neurofibrillary degeneration' OR 'alzheimer neuron degeneration' OR 'alzheimer perusini disease' OR 'alzheimer sclerosis' OR 'alzheimer syndrome' OR 'alzheimer`s disease' OR 'cortical sclerosis, diffuse' OR 'dementia, alzheimer' OR 'diffuse cortical sclerosis' OR 'late onset alzheimer disease' OR 'dementia'/exp OR 'amentia' OR 'dementia' OR 'demention' OR 'mild cognitive impairment'/exp OR 'amnestic mild cognitive impairment' OR 'mild cognitive impairment' OR 'cognitive defect'/exp OR 'cognition disorder' OR 'cognition disorders' OR 'cognitive defect' OR 'cognitive defects' OR 'cognitive deficit' OR 'cognitive disability' OR 'cognitive disorder' OR 'cognitive disorders' OR 'cognitive impairment' OR 'delirium, dementia, amnestic, cognitive disorders' OR 'overinclusion' OR 'response interference' OR 'cognition'/exp OR 'cognition' OR 'cognitive accessibility'OR 'cognitive balance' OR 'cognitive dissonance' OR 'cognitive function' OR 'cognitive structure' OR 'cognitive symptoms' OR 'cognitive task' OR 'cognitive thinking' OR 'neurobehavioural manifestations' OR 'volition' OR 'memory'/exp OR 'item recall' OR 'memory' OR 'memory function' OR 'nonspatial memory' OR 'remembering' OR 'reminiscence' OR 'mental capacity'/exp OR 'ability, mental' OR 'attainment' OR 'capacity, mental' OR 'fitness, mental' OR 'mental ability' OR 'mental capacity' OR 'mental competency' OR ad OR mci OR 'cognitive fail*' OR 'cognitive decline*' OR 'cognitive impair*' OR alzheimer* OR dement* OR 'cognitive dysfunction' OR 'cognitive performance' OR cognitive) AND ('probiotic agent'/exp OR 'probiotic' OR 'probiotic agent' OR 'probiotics' OR 'yeast'/exp OR 'flora, yeast' OR 'fungi, yeast' OR 'yeast' OR 'yeast fermentation' OR 'yeast flora' OR 'yeast fungus' OR 'yeast germination' OR 'yeast metabolism' OR 'yeasts' OR 'yoghurt'/exp OR 'yoghourt' OR 'yoghurt' OR 'yogurt' OR 'zabadi' OR 'lactobacillus'/exp OR 'betabacterium' OR 'lactobacileae' OR 'lactobacilleae' OR 'lactobacillus' OR 'lactobacteria' OR 'lactobacilli' OR 'bifidobacterium'/exp OR 'bifidobacterium' OR 'fermented dairy product'/exp OR 'cultured dairy foods' OR 'cultured dairy product' OR 'cultured milk foods' OR 'cultured milk product' OR 'cultured milk products' OR 'fermented dairy foods' OR 'fermented dairy product' OR 'fermented milk' OR 'fermented milk product' OR 'fermented product'/exp OR 'fermented food' OR 'fermented foods' OR 'fermented product' OR 'synbiotic agent'/exp OR 'synbiotic' OR 'synbiotic agent' OR 'synbiotics' OR probiotic*) AND ('placebo'/exp OR 'placebo' OR 'placebo gel' OR 'placebos') AND ('randomized controlled trial'/exp OR 'controlled trial, randomized' OR 'randomised controlled study' OR 'randomised controlled trial' OR 'randomized controlled study' OR 'randomized controlled trial' OR 'trial, randomized controlled' OR 'controlled clinical trial'/exp OR 'clinical trial, controlled' OR 'controlled clinical comparison' OR 'controlled clinical drug trial' OR 'controlled clinical experiment' OR 'controlled clinical

study' OR 'controlled clinical test' OR 'controlled clinical trial' OR rct OR random* OR control* OR trial*) AND 'human'/de AND ([adult]/lim OR [aged]/lim OR [middle aged]/lim OR [very elderly]/lim OR [young adult]/lim)

PubMed

Search strategy: (((("Adult"[Mesh]) AND "Humans"[Mesh]) AND ((((((((((((((((("Alzheimer Disease"[Mesh]) OR Alzheimer*) OR AD) OR "Dementia"[Mesh]) OR Dement*) OR mild cognitive impairment) OR MCI) OR ("Cognition"[Mesh] OR "Cognition Disorders"[Mesh])) OR ("Memory"[Mesh] OR "Memory Disorders"[Mesh])) OR "Cognitive Dysfunction"[Mesh]) OR cognitive defect) OR Mental capacity) OR Cognitive) OR cognitive performance) OR cognitive impair*) OR cognitive decline*) OR cognitive fail*)) AND ("Placebos"[Mesh]) AND (((((((((("Probiotics"[Mesh]) OR "Cultured Milk Products"[Mesh]) OR "Yogurt"[Mesh]) OR (("Yeast, Dried"[Mesh]) OR "Yeasts"[Mesh])) OR "Bifidobacterium"[Mesh]) OR "Lactobacillus"[Mesh]) OR "Synbiotics"[Mesh]) OR probiotic*) OR fermented product) OR fermented dairy product)) AND (((((((("Randomized Controlled Trial" [Publication Type] OR "Controlled Clinical Trial" [Publication Type])) OR randomized controlled trials) OR Controlled Clinical Trials) OR RCT) OR random*) OR control*) OR trial*)))))

Cochrane Central Register of Controlled Trials (CENTRAL)

Search strategy:

#1 MeSH descriptor: [Lactobacillus] explode all trees
#2 MeSH descriptor: [Yogurt] explode all trees
#3 MeSH descriptor: [Probiotics] explode all trees
#4 MeSH descriptor: [Bifidobacterium] explode all trees
#5 MeSH descriptor: [Yeasts] explode all trees
#6 MeSH descriptor: [Cultured Milk Products] explode all trees
#7 MeSH descriptor: [Synbiotics] explode all trees
#8 #1 or #2 or #3 or #4 or #5 or #6 or #7
#9 "probiotic*" or "fermented product" or "fermented dairy product"
#10 #8 or #9
#11 adult and humans
#12 MeSH descriptor: [Alzheimer Disease] explode all trees
#13 MeSH descriptor: [Dementia] explode all trees
#14 MeSH descriptor: [Cognitive Dysfunction] explode all trees
#15 MeSH descriptor: [Cognition] explode all trees
#16 MeSH descriptor: [Memory] explode all trees
#17 MeSH descriptor: [Cognition Disorders] explode all trees
#18 MeSH descriptor: [Memory Disorders] explode all trees
#19 Alzheimer* or AD or Dement* or "cognitive" or "mild cognitive impairment" or MCI or "cognitive fail*" or "cognitive performance" or "cognitive impair*" or "cognitive decline*" or "memory function" or "Mental capacity" or "cognitive defect"
#20 #12 or #13 or #14 or #15 or #16 or #17 or #18 or #19
#21 MeSH descriptor: [Randomized Controlled Trial] explode all trees
#22 MeSH descriptor: [Controlled Clinical Trial] explode all trees
#23 RCT or "randomized controlled trials" or "Controlled Clinical Trials" or "random*" or "control*" or "trial*"
#24 #21 or #22 or #23
#25 #10 and #20 and #24
#26 #25 and trails

Web of Science

Search strategy: ('alzheimer disease'/exp OR 'alzeimer disease' OR 'alzeimer`s disease' OR 'alzeimers disease' OR 'alzheimer dementia' OR 'alzheimer disease' OR 'alzheimers disease' OR 'alzheimer fibrillary change' OR 'alzheimer fibrillary lesion'OR 'alzheimer neurofibrillary change' OR 'alzheimer neurofibrillary degeneration' OR 'alzheimer neuron degeneration' OR 'alzheimer perusini disease' OR 'alzheimer sclerosis' OR 'alzheimer syndrome' OR 'alzheimer`s disease' OR 'cortical sclerosis, diffuse' OR 'dementia, alzheimer' OR 'diffuse cortical sclerosis' OR 'late onset alzheimer disease' OR 'dementia'/exp OR 'amentia' OR 'dementia' OR 'demention' OR 'mild cognitive impairment'/exp OR 'amnestic mild cognitive impairment' OR 'mild cognitive impairment' OR 'cognitive defect'/exp OR 'cognition disorder' OR 'cognition disorders' OR 'cognitive defect' OR 'cognitive defects' OR 'cognitive deficit' OR 'cognitive disability' OR 'cognitive disorder' OR 'cognitive disorders' OR 'cognitive impairment' OR 'delirium, dementia, amnestic, cognitive disorders' OR 'overinclusion' OR 'response interference' OR 'cognition'/exp OR 'cognition' OR 'cognitive accessibility'OR 'cognitive balance' OR 'cognitive dissonance' OR 'cognitive function' OR 'cognitive structure' OR 'cognitive symptoms' OR 'cognitive task' OR 'cognitive thinking' OR 'neurobehavioural manifestations' OR 'volition' OR 'memory'/exp OR 'item recall' OR 'memory' OR 'memory function' OR 'nonspatial memory' OR 'remembering' OR 'reminiscence' OR 'mental capacity'/exp OR 'ability, mental' OR 'attainment' OR 'capacity, mental' OR 'fitness, mental' OR 'mental ability' OR 'mental capacity' OR 'mental competency' OR ad OR mci OR 'cognitive fail*' OR 'cognitive decline*'

OR 'cognitive impair*' OR alzheimer* OR dement* OR 'cognitive dysfunction' OR 'cognitive performance' OR cognitive) AND ('probiotic agent'/exp OR 'probiotic' OR 'probiotic agent' OR 'probiotics' OR 'yeast'/exp OR 'flora, yeast' OR 'fungi, yeast' OR 'yeast' OR 'yeast fermentation' OR 'yeast flora' OR 'yeast fungus' OR 'yeast germination' OR 'yeast metabolism' OR 'yeasts' OR 'yoghurt'/exp OR 'yoghourt' OR 'yoghurt' OR 'yogurt' OR 'zabadi' OR 'lactobacillus'/exp OR 'betabacterium' OR 'lactobacileae' OR 'lactobacilleae' OR 'lactobacillus' OR 'lactobacteria' OR 'lactobacilli' OR 'bifidobacterium'/exp OR 'bifidobacterium' OR 'fermented dairy product'/exp OR 'cultured dairy foods' OR 'cultured dairy product' OR 'cultured milk foods' OR 'cultured milk product' OR 'cultured milk products' OR 'fermented dairy foods' OR 'fermented dairy product' OR 'fermented milk' OR 'fermented milk product' OR 'fermented product'/exp OR 'fermented food' OR 'fermented foods' OR 'fermented product' OR 'synbiotic agent'/exp OR 'synbiotic' OR 'synbiotic agent' OR 'synbiotics' OR probiotic*) AND ('randomized controlled trial'/exp OR 'controlled trial, randomized' OR 'randomised controlled study' OR 'randomised controlled trial' OR 'randomized controlled study' OR 'randomized controlled trial' OR 'trial, randomized controlled' OR 'controlled clinical trial'/exp OR 'clinical trial, controlled' OR 'controlled clinical comparison' OR 'controlled clinical drug trial' OR 'controlled clinical experiment' OR 'controlled clinical study' OR 'controlled clinical test' OR 'controlled clinical trial' OR rct OR random* OR control* OR trial*) AND (Human AND adult)

Study records

Data management

All electronic databases citations retrieved using the above search strategy will be imported into Endnote (Endnote X6, Thomson Reuters, San Francisco, CA) to manage and delete duplicate records. Studies retrieved from reference lists of published reviews and retrieved articles will be entered into Microsoft Excel spread sheet for de-duplication and screening.

Selection process

Screening will be performed by screening the titles and abstracts followed by the retrieval and screening of full text articles using the inclusion and exclusion criteria. We will contact the study authors for full text articles with the maximum of three attempts for any articles that we are unable to retrieve. Articles were initially and independently screened for eligibility by two investigators. Any disagreements that arise between the reviewers will be resolved through discussion, or with a third reviewer. Moreover, duplicate and non-eligible articles will be excluded and reasons for exclusion will be recorded. A PRISMA flow chart will be used to trace the overall process.

Data collection process

Two reviewers will extract data from the selected studies independently and crosscheck all articles for accuracy. Disagreements will be resolved by discussion between the reviewers or with a third reviewer.

Data items

We will extract data according to our predetermined form(as shown in Supplementary Table 1), containing five main categories: 1) Basic information of the included studies (e.g., study title, name of first author, year of publication, whether single-center or multicenter, country of study, total sample size); 2) Characteristics of the participants (e.g., gender, age, disease type, diagnostic criteria); 3) Intervention-related variables (e.g., type of strains, dosage, duration); 4) Outcome measurements (e.g., MMSE, TAC, GSH, MDA, hs-CRP, NO); 5) Main findings of each included studies. To perform the meta-analysis, the following data form of outcome assessments are to be extracted: the mean change score along with the associated variance (standard deviation [SD] or standard error of the mean [SEM]). When change scores are not available, the scores (mean ± SD or mean ± SEM) and the number of participants at baseline and post-intervention will be extracted.

Outcomes and prioritization

Primary outcomes

The primary outcomes of this study are the standardized mean differences (SMDs) of change from baseline between probiotics and placebo group, including pooled estimate of cognition, malondialdehyde (MDA), high-sensitivity C-reactive protein (hs-CRP), total glutathione (GSH) and nitric oxide (NO).

Secondary outcomes

Where available, secondary outcomes for this review will include subgroups such as cognitive rating scales (MMSE versus non-MMSE), disease type (AD versus MCI), strains of flora (multiple versus sole).

Risk of bias in individual studies

An assessment of risk of bias will be incorporated into our analysis. By assessing the quality of the studies that will be included in the meta-analysis, we can assess the strength of the body of evidence. In particular, information relating to bias will be extracted from each study during the data extraction process. This assessment will follow the same procedure with the data collection process where disagreements will be resolved by

discussion between the reviewers or with a third reviewer. To facilitate the appraisal of possible risk of bias, paired reviewers will evaluate independently the risk of bias of included RCTs using the tool of the Cochrane Handbook for Systematic Reviews of Interventions, containing the following six criteria: random sequence generation, allocation concealment, blindness of participants and personnel, blindness of outcome assessment, incomplete outcome data, selective reporting, and other sources of bias. Furthermore, to evaluate the risk of bias, reviewers will assess and rate each of the above items into three ratings: "low risk of bias" or "high risk of bias" or "uncertain-risk". In particular, "low risk" indicates that further research is very unlikely to change the confidence of the estimates whereas "high risk" indicates that further research is very likely to change the estimate. The results of the risk of bias assessment will be pooled into Revman 5.3, and generate a "summary of risk of bias assessment" table.

Data synthesis

We will use STATA software (version 12; StataCorp) to perform the statistical analyses. The primary outcomes of this study are the standardized mean differences (SMDs) of change from baseline between probiotics and placebo group. The SMDs will be tested by a Z statistic, and a two-tailed $P < 0.05$ will be regarded as statistically significant. To determine the extent of variation between the selected studies, tests of heterogeneity will be performed. The inter-study heterogeneity was examined by chi-square (χ^2) statistics and I^2 statistics. Higgins and Colleagues provided tentative benchmarks for I^2 where values below 25% might be considered as low, 50-75% as moderate and above 75% as high. In our study, the heterogeneity among the different studies was considered high if $P < 0.1$ for the χ^2 statistic or $I^2 > 50\%$ [22]. A random-effects model was used if significant heterogeneity was shown among trials. Otherwise, the results were obtained from a fixed-effects model. Subgroup analyses will be performed to examine the possible source of heterogeneity within these studies.

Meta-bias(es)

We will use STATA software (version 12; StataCorp) to perform sensitivity analysis and publication bias. A sensitivity analysis will be conducted to test the reliability of the findings using the leave-one-out method, while publication bias will be assessed by Egger's test and Begg's test.

AUTHOR CONTRIBUTIONS

(1) Research planning and design: Mingliang Chen and Haoyue Deng; (2) Feasibility testing of the study: Mingliang Chen, Haoyue Deng, Xunhu Dong and Zhongmin Zou; (3) Conceptualization: Mingliang Chen and Haoyue Deng; (4) Methodology: Mingliang Chen, Haoyue Deng, Xunhu Dong; (5) Writing – original draft: Haoyue Deng; (6) Writing – review & editing: Mingliang Chen, Zhongmin Zou; (7) Supervision: Mingliang Chen, Zhongmin Zou.

FUNDING

(1) National Natural Science Foundation of China (Grant number : 81703268); (2) National Postdoctoral Foundation of China (Grant number : 2019M663973); (3) National Postdoctoral Program for Innovative Talents (Grant number : BX20180378); (4) The Excellent Youth Foundation of the Third Military Medical University (2017).

REFERENCES

1. ADI. World Alzheimer Report 2018: The state of the art of dementia research: New frontiers. An Analysis of Prevalence, Incidence, Cost and Trends. London: Alzheimer's Disease International; 2018.

2. Gauthier S, Reisberg B, Zaudig M, Petersen RC, Ritchie K, Broich K, Belleville S, Brodaty H, Bennett D, Chertkow H, Cummings JL, de Leon M, Feldman H, et al, and International Psychogeriatric Association Expert Conference on mild cognitive impairment. Mild cognitive impairment. Lancet. 2006; 367:1262–70. https://doi.org/10.1016/S0140-6736(06)68542-5 PMID:16631882

3. Manayi A, Saeidnia S, Gohari AR, Abdollahi M. Methods for the discovery of new anti-aging products—targeted approaches. Expert Opin Drug Discov. 2014; 9:383–405. https://doi.org/10.1517/17460441.2014.885014 PMID:24494592

4. La Rosa F, Clerici M, Ratto D, Occhinegro A, Licito A, Romeo M, Iorio CD, Rossi P. The Gut-Brain Axis in Alzheimer's Disease and Omega-3. A Critical Overview of Clinical Trials. Nutrients. 2018; 10. https://doi.org/10.3390/nu10091267 PMID:30205543

5. Pistollato F, Sumalla Cano S, Elio I, Masias Vergara M, Giampieri F, Battino M. Role of gut microbiota and nutrients in amyloid formation and pathogenesis of Alzheimer disease. Nutr Rev. 2016; 74:624–34. https://doi.org/10.1093/nutrit/nuw023 PMID:27634977

6. Mayer EA, Tillisch K, Gupta A. Gut/brain axis and the microbiota. J Clin Invest. 2015; 125:926–38. https://doi.org/10.1172/JCI76304 PMID:25689247

7. Liang S, Wang T, Hu X, Luo J, Li W, Wu X, Duan Y, Jin F.

Administration of Lactobacillus helveticus NS8 improves behavioral, cognitive, and biochemical aberrations caused by chronic restraint stress. Neuroscience. 2015; 310:561–77. https://doi.org/10.1016/j.neuroscience.2015.09.033 PMID:26408987

8. Leblhuber F, Egger M, Schuetz B, Fuchs D. Commentary: effect of probiotic supplementation on cognitive function and metabolic status in Alzheimer's disease: a randomized, double-blind and controlled trial. Front Aging Neurosci. 2018; 10:54. https://doi.org/10.3389/fnagi.2018.00054 PMID:29559906

9. Bostancikloğlu M. The role of gut microbiota in pathogenesis of Alzheimer's disease. J Appl Microbiol. 2019. https://doi.org/10.1111/jam.14264

10. Kowalski K, Mulak A. Brain-Gut-Microbiota Axis in Alzheimer's Disease. J Neurogastroenterol Motil. 2019; 25:48–60. https://doi.org/10.5056/jnm18087 PMID:30646475

11. Lynch SV, Pedersen O. The Human Intestinal Microbiome in Health and Disease. N Engl J Med. 2016; 375:2369–79. https://doi.org/10.1056/NEJMra1600266 PMID:27974040

12. Chunchai T, Thunapong W, Yasom S, Wanchai K, Eaimworawuthikul S, Metzler G, Lungkaphin A, Pongchaidecha A, Sirilun S, Chaiyasut C, Pratchayasakul W, Thiennimitr P, Chattipakorn N, Chattipakorn SC. Decreased microglial activation through gut-brain axis by prebiotics, probiotics, or synbiotics effectively restored cognitive function in obese-insulin resistant rats. J Neuroinflammation. 2018; 15:11. https://doi.org/10.1186/s12974-018-1055-2 PMID:29316965

13. Wallace CJ, Milev R. The effects of probiotics on depressive symptoms in humans: a systematic review. Ann Gen Psychiatry. 2017; 16:14. https://doi.org/10.1186/s12991-017-0138-2 PMID:28239408

14. Sharon G, Sampson TR, Geschwind DH, Mazmanian SK. The Central Nervous System and the Gut Microbiome. Cell. 2016; 167:915–32. https://doi.org/10.1016/j.cell.2016.10.027 PMID:27814521

15. Wang H, Lee IS, Braun C, Enck P. Effect of Probiotics on Central Nervous System Functions in Animals and Humans: A Systematic Review. J Neurogastroenterol Motil. 2016; 22:589–605. https://doi.org/10.5056/jnm16018 PMID:27413138

16. Akbari E, Asemi Z, Daneshvar Kakhaki R, Bahmani F, Kouchaki E, Tamtaji OR, Hamidi GA, Salami M. Effect of Probiotic Supplementation on Cognitive Function and Metabolic Status in Alzheimer's Disease: A Randomized, Double-Blind and Controlled Trial. Front Aging Neurosci. 2016; 8:256. https://doi.org/10.3389/fnagi.2016.00256 PMID:27891089

17. Tamtaji OR, Heidari-Soureshjani R, Mirhosseini N, Kouchaki E, Bahmani F, Aghadavod E, Tajabadi-Ebrahimi M, Asemi Z. Probiotic and selenium co-supplementation, and the effects on clinical, metabolic and genetic status in Alzheimer's disease: A randomized, double-blind, controlled trial. Clin Nutr. 2019; 38:2569–75. https://doi.org/10.1016/j.clnu.2018.11.034 PMID:30642737

18. Agahi A, Hamidi GA, Daneshvar R, Hamdieh M, Soheili M, Alinaghipour A, Esmaeili Taba SM, Salami M. Does Severity of Alzheimer's Disease Contribute to Its Responsiveness to Modifying Gut Microbiota? A Double Blind Clinical Trial. Front Neurol. 2018; 9:662. https://doi.org/10.3389/fneur.2018.00662 PMID:30158897

19. Kobayashi Y, Kuhara T, Oki M, Xiao JZ. Effects of *Bifidobacterium breve* A1 on the cognitive function of older adults with memory complaints: a randomised, double-blind, placebo-controlled trial. Benef Microbes. 2019; 10:511–20. https://doi.org/10.3920/BM2018.0170 PMID:31090457

20. Hwang YH, Park S, Paik JW, Chae SW, Kim DH, Jeong DG, Ha E, Kim M, Hong G, Park SH, Jung SJ, Lee SM, Na KH, et al. Efficacy and Safety of *Lactobacillus Plantarum* C29-Fermented Soybean (DW2009) in Individuals with Mild Cognitive Impairment: A 12-Week, Multi-Center, Randomized, Double-Blind, Placebo-Controlled Clinical Trial. Nutrients. 2019; 11:E305. https://doi.org/10.3390/nu11020305 PMID:30717153

21. Cheng LH, Liu YW, Wu CC, Wang S, Tsai YC. Psychobiotics in mental health, neurodegenerative and neurodevelopmental disorders. Yao Wu Shi Pin Fen Xi. 2019; 27:632–48. https://doi.org/10.1016/j.jfda.2019.01.002 PMID:31324280

22. Higgins J, Green S. Cochrane Handbook for Systematic Reviews of Interventions, Version 5.1.0. 2013.
