# Supplementary material for: Efficacy of probiotics on cognition, and biomarkers of inflammation and oxidative stress in adults with Alzheimer’s disease or mild cognitive impairment — a meta-analysis of randomized controlled trials
Source: Aging (Albany NY). 2020 Feb 15;12(4):4010–39. doi: 10.18632/aging.102810 (PMC7066922; doi:10.18632/aging.102810)
Supplement: Supplementary Table 1 [file aging-12-102810-s001..docx]

SUPPLEMENTARY TABLE
Supplementary Table 1. Main characteristics of the included studies

| **No.** | **Basic information** | | | | | | **Participants** | | | | | **Intervention** | | | | **Outcome measurements**  **(Cognitive outcomes/Inflammatory biomarkers/oxidative biomarkers)** | | | | | | | **Main findings** |
| --- | --- | --- | --- | --- | --- | --- | --- | --- | --- | --- | --- | --- | --- | --- | --- | --- | --- | --- | --- | --- | --- | --- | --- |
|  | **Study** | **First author** | **Year** | **Sample size** | **Study location** | **Study design** | **Type** | **M/F** | | **Age** | | **Type of strains** | **Duration** | **Dose (mg/day)** | **CON** | **Outcomes** | **PRO** | | | **CON** | | |  |
|  |  |  |  |  |  |  |  | **CON** | **PRO** | **CON** | **PRO** |  |  |  |  |  | **Baseline 1 (N1)** | **Final 1 (N1')** | **Change from baseline 1** | **Baseline 2 (N2)** | **Final 2 (N2')** | **Change from baseline 2** |  |
|  |  |  |  |  |  |  |  |  |  |  |  |  |  |  |  |  |  |  |  |  |  |  |  |
|  |  |  |  |  |  |  |  |  |  |  |  |  |  |  |  |  |  |  |  |  |  |  |  |
|  |  |  |  |  |  |  |  |  |  |  |  |  |  |  |  |  |  |  |  |  |  |  |  |
|  |  |  |  |  |  |  |  |  |  |  |  |  |  |  |  |  |  |  |  |  |  |  |  |
|  |  |  |  |  |  |  |  |  |  |  |  |  |  |  |  |  |  |  |  |  |  |  |  |
|  |  |  |  |  |  |  |  |  |  |  |  |  |  |  |  |  |  |  |  |  |  |  |  |
|  |  |  |  |  |  |  |  |  |  |  |  |  |  |  |  |  |  |  |  |  |  |  |  |
|  |  |  |  |  |  |  |  |  |  |  |  |  |  |  |  |  |  |  |  |  |  |  |  |
|  |  |  |  |  |  |  |  |  |  |  |  |  |  |  |  |  |  |  |  |  |  |  |  |

Abbreviations: M, male; F, female; PRO, probiotics group; CON, control group
